# Profile of Polyphenol Compounds of Five Muscadine Grapes Cultivated in the United States and in Newly Adapted Locations in China

**DOI:** 10.3390/ijms18030631

**Published:** 2017-03-14

**Authors:** Zheng Wei, Jianming Luo, Yu Huang, Wenfeng Guo, Yali Zhang, Huan Guan, Changmou Xu, Jiang Lu

**Affiliations:** 1 College of Food Science and Nutritional Engineering, China Agricultural University, Beijing 100083, China; weizheng76096@sina.com (Z.W.); baiding86@126.com (J.L.); zhangyali@cau.edu.cn (Y.Z.); 2 Guangxi Crop Genetic Improvement and Biotechnology Laboratory, Guangxi Academy of Agricultural Sciences, Nanning 530007, China; gwenfeng@163.com (W.G.); guanhuan2010@163.com (H.G.); 3 Grape and Wine Research Institute, Guangxi Academy of Agricultural Sciences, Nanning 530007, China; hy0611@163.com; 4 Department of Food Science and Technology, University of Nebraska-Lincoln, Lincoln, NE 68508, USA; cxu13@unl.edu; 5 Center for Viticulture and Enology, School of Agriculture and Biology, Shanghai Jiao Tong University, Shanghai 200240, China

**Keywords:** polyphenols, ellagic acids, muscadine grapes, geographical location, UPLC-Triple TOF-MS/MS

## Abstract

Polyphenol compositions and concentrations in skins and seeds of five muscadine grapes (cv. “Noble”, “Alachua”, “Carlos”, “Fry”, and “Granny Val”) cultivated in the United States (Tallahassee-Florida, TA-FL) and South China (Nanning-Guangxi, NN-GX and Pu’er-Yunnan, PE-YN) were investigated, using ultra performance liquid chromatography tandem triple quadrupole time-of-flight mass spectrometry (UPLC Triple TOF MS/MS). Fourteen ellagitannins were newly identified in these muscadine grapes. The grapes grown in NN-GX accumulated higher levels of ellagic acid, methyl brevifolin carboxylate, and ellagic acid glucoside in skins, and penta-*O*-galloyl-glucose in seeds. In PE-YN, more flavonols were detected in skins, and higher contents of flavan-3-ols, ellagic acid, and methyl gallate were identified in seeds. Abundant seed gallic acid and flavonols were found among the grapes grown in TA-FL. Based on principal component analysis (PCA) of 54 evaluation parameters, various cultivars grown in different locations could be grouped together and vice versa for the same cultivar cultivated in different regions. This is the result of the interaction between genotype and environmental conditions, which apparently influences the polyphenol synthesis and accumulation.

## 1. Introduction

*Muscadinia rotundifolia* Michx., commonly called as muscadine grape, is a genus from the Vitaceae family. Muscadine grapes are indigenous to the southeast United States and are well-adapted to the warm, humid environment [1,2]. They have important economic value, mainly due to their resistance to Pierce’s disease (*Xylella fastidiosa*), *Plasmopara viticola*, and *Elsinoe ampelina* [3,4,5,6]. They also have multiple health benefits, including antioxidant, antiviral agents, anticancer, antibacterial, and anti-inflammatory properties [7,8,9]. The muscadine grape currently comprises over 100 cultivars such as “Noble”, “Alachua”, “Carlos”, “Fry”, and “Granny Val”, among many others [2]. All commercial muscadine grape production occurs in the United States [7,10]. Recently, some of these muscadine grape cultivars have been introduced to South China for the first time. These cultivars provide better possibilities, adapting to local climates for table and wine grape production in areas unsuitable for the growth of *Vitis vinifera* [11,12].

Polyphenol compounds are secondary metabolites and represent the third most abundant constituent in grapes after carbohydrates and fruit acids. They are mainly distributed in seeds (60%–70%) and skins (28%–35%), with less than 10% in pulp [5,13]. Major polyphenols present in muscadine grapes have been previously reported. For example, the skins and seeds of these grapes contained large amounts of ellagic acids (ellagic acid derivatives and ellagitannins) [13,14]. The anthocyanin levels in red skins of “Noble” and “Alachua” ranged from 292.0 to 554.9 mg as malvidin-3-glucoside per 100 g dry weight (DM) [12]. In addition, their skins also had flavonols such as glycosides of quercetin, kaempferol, and myricetin [3,5]. Meanwhile, abundant flavan-3-ols like procyanidins (epicatechin and epicatechin gallate) and condensed tannins were identified in muscadine seeds [13,15,16]. However, these identifications have not been fully elucidated, and the contents varied greatly in different reports [1,10,13,14,17]. These differences were mainly because of the complexity, diversity, and polymeric and isomeric forms of large size of these compounds, as well as the lack of complete fragmentation data and suitable quantitative standards [18,19]. In addition, materials from different growing conditions may also contribute to the variance.

The synthesis and accumulation of polyphenol compounds in fruits are regulated by genetic and climatic conditions, as well as agricultural practices [20]. Marshall et al. [5] examined the polyphenol concentrations in muscadine fruits of 21 cultivars, and found that the stilbene, ellagic acid, and flavonol differed significantly among cultivars. Chen [7] investigated skin extracts among 17 muscadine cultivars (6 bronze and 11 dark) and discovered that dark-skinned cultivars had higher contents than the bronze ones with respect to polyphenol and ellagic acids. Meanwhile, the impact of climatic factors on polyphenol compositions and concentrations of grape berries has been widely studied. It was reported that these compounds of grape berries varied in different vineyards, and variations were also presented among cultivars being grown in the same vineyard for two consecutive years [21]. Additionally, agronomic strategies such as alteration of environmental conditions (light, temperature, mineral nutrition, and water management), application of elicitors, stimulating agents and plant activators have been employed to enhance the biosynthesis of polyphenols [18,22,23,24,25].

To our knowledge, the muscadine grapes have never been reported as growing commercially in countries outside the United States. Therefore, the aim of this study is to characterize and distinguish the polyphenol compositions of five muscadine grape cultivars grown in China (Nanning-Guangxi and Pu’er-Yunnan) and the native United States (Tallahassee-Florida), respectively, and to link the differences to the growing conditions in different geographical regions in order to elucidate factors related to the polyphenol accumulation. The overall goal of this study is to develop a strategy to grow muscadine grapes with richer polyphenol compositions in China.

## 2. Results

### 2.1. Total Phenolic Content (TPC)

Muscadine grapes cv. “Noble”, “Alachua”, “Carlos”, and “Fry” harvested in 2012 season from Nanning-Guangxi (NN-GX) and Pu’er-Yunnan (PE-YN), China, had higher total phenolic content (TPC) in skins and lower in seeds than those grown in their native origin of Tallahassee-Florida (TA-FL), United States (Table 1). “Granny Val”, as an exception, had less TPC in skins but was richer in TPC in its seeds in China than in the USA. Nevertheless, the combined TPCs of the muscadine grape skins and seeds grown in China were a little bit lower than those of grapes grown in the USA. For all cultivars grown in China and USA, TPCs of grape seeds were about 3-fold higher than those of skins. For example, the TPC in “Noble” seeds was 96.65 mg gallic acid equivalent (GAE)/g dry weight (DW), while in skins it was 38.96 mg GAE/g DW. This is because the precursors of ellagic acid biosynthesis and condensed tannins were mainly detected in seeds, which resulting a higher TPC in seeds than in skins.

### 2.2. Polyphenol Composition and Accumulation

#### 2.2.1. Ellagic Acids and Precursors Profiles

Ellagic acids (ellagic acid derivatives and ellagitannins) and their precursors (gallic acid derivatives) were the chief polyphenol compounds identified in muscadine skins and seeds (Table 2). In 2012 season, the grapes grown in China exhibited significantly higher contents of ellagic acids and precursors than those grown in the USA, except “Noble”, which possessed significantly lower levels of ellagic acids and precursors in seeds. The highest values of skin ellagic acids and precursors were observed in cultivars “Granny Val” and “Alachua” from NN-GX (248.73 and 220.99 mg GAE/100 g DW, respectively), whereas the lowest were found in cultivar “Granny Val” from TA-FL (85.42 mg GAE/100 g DW). The seeds of red cultivars from TA-FL and NN-GX exhibited higher contents of ellagic acids and precursors than those in PE-YN. For instance, “Noble” from TA-FL had 636.75 mg GAE/100 g DW, while this value was 380.61 mg GAE/100 g DW in PE-YN.

**Figure 1:**
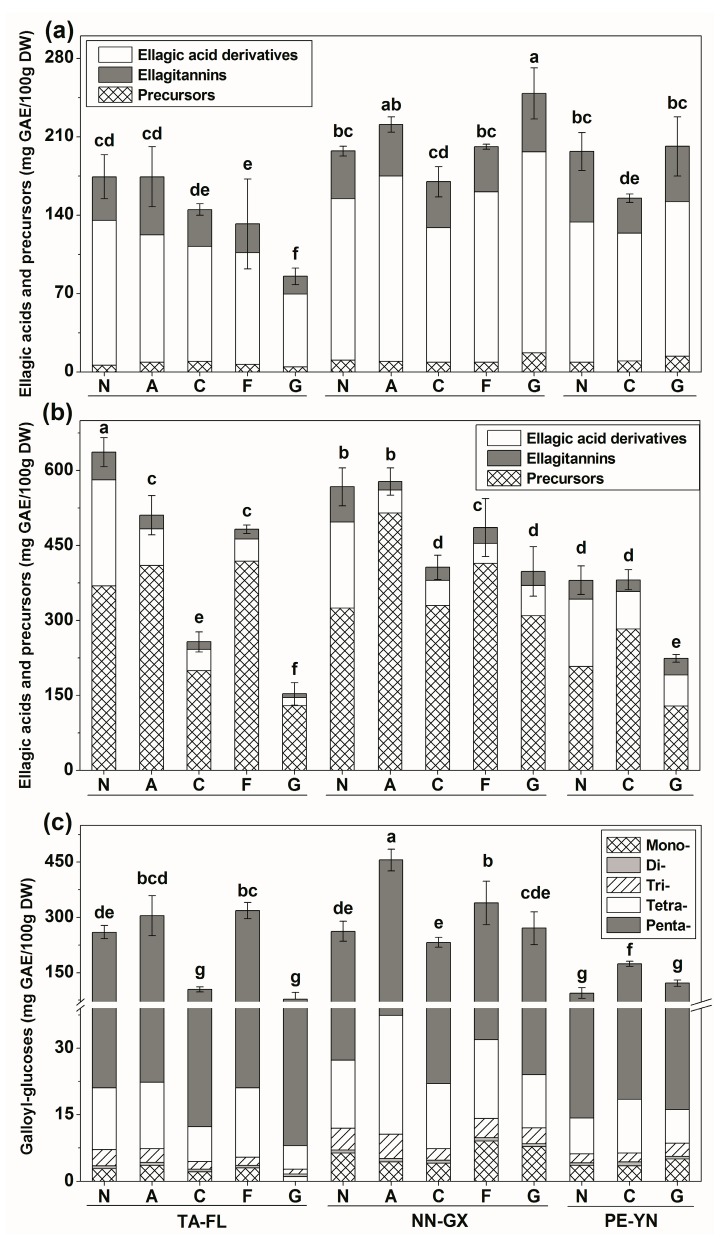
Ellagic acids and precursors distribution in five muscadine skins (**a**) and seeds (**b**), and galloyl-glucoses distribution in muscadine seeds (**c**), among three different regions in the 2012 season. On the top of each column, standard deviation is show for the total content, and different small letters indicate significant differences (Duncan’s test, *p* = 0.05). See Table 1 for the abbreviation of cultivars and regions.

A total of 46 different ellagic acids and precursors were identified in 2012’s skin samples, whereas only 33 different kinds were detected in seeds (Tables S1–S3). These compounds varied among muscadine grape cultivars. The “Noble” had more ellagic acids and precursors in both skins and seeds than the other cultivars. For example, there were 40 different compounds in skins and 26 in seeds of “Noble” grape grown in PE-YN. The constituents of ellagic acids and precursors differed between the skins and seeds. For example, the ellagic acid derivatives were the primary form found in skins (Figure 1a), while the precursors were the main type detected in seeds (Figure 1b). Significant content variations of precursors were found among cultivars growing in different regions. For example, “Alachua” from NN-GX contained the highest level of seed precursors (514.99 mg GAE/100 g DW), whereas “Granny Val” from TA-FL had the lowest (129.29 mg GAE/100 g DW).

Skin ellagic acid accounted for over 55% of total ellagic acids and precursors in all of the muscadine grapes grown in China and USA (Tables S1–S3). Methyl brevifolin carboxylate and tri-*O*-methyl ellagic acid were common in the grapes from TA-FL and NN-GX, whereas ducheside A and B were identified mostly from PE-YN. Ellagic acid glucoside and diglucoside were both detected in skins, and the glucoside/diglucoside ratio was higher in NN-GX.

Thirteen unknown ellagitannins with *m*/*z* 443 to 957 were first reported in muscadine skins from this study. Among them, ellagitannin *m*/*z* 643 was the most common one, particularly in “Noble” grapes grown in PE-YN. In addition, mono-*O*-methy ellagic acid, ellagitannin *m*/*z* 681 and 689 were almost exclusively found in red cultivars, yet tri-*O*-galloyl-glucose was only detected in bronze ones among samples from all the growing regions.

Interestingly, no tetra- and penta-*O*-galloyl-glucoses were detected in the muscadine skins, while five galloyl-glucoses (from mono- to penta-*O*-) were identified in the seeds, of which the overall sum of the five galloyl-glucoses accounted for above 50% of the total seed ellagic acids and precursors in the three regions (Figure 1c). Additionally, muscadine grapes in NN-GX demonstrated the highest content of penta-*O*-galloyl-glucose, while the grapes in TA-FL exhibited higher levels of gallic acid, and methyl gallate was dominant among the muscadine grapes produced in PE-YN. Moreover, an unkown ellagitannin *m*/*z* 967 was quantified in muscadine seeds for the first time.

#### 2.2.2. Flavonols Profiles

Flavonols were the second-highest polyphenol in terms of content detected in muscadine skins in our study, whereas their contents in seeds were relatively low (Table 2). The cultivar “Noble” from PE-YN and “Fry” from NN-GX were characterized with significantly higher skin flavonols (38.92 and 38.89 mg rutin equivalent (RE)/100 g DW, respectively) than others. However, the seed flavonols significantly differed among the growing regions. In general, higher flavonols were found among grape cultivars grown in the USA. (10.17–27.89 mg RE/100 g DW in TA-FL) than in China (10.48–14.47 mg RE/100 g DW in PE-YN, and 7.03–10.52 mg RE/100 g DW in NN-GX).

The contents of skin flavonol glycosides ranked in order as following: aglycones > monoglucosides > acylates > diglucosides, according to the concentrations (Tables S1–S3). The region of PE-YN exhibited significantly higher contents of aglycones in comparison to other regions. Additionally, the monoglucosides (glucoside and glucuronide) were richer in red cultivars than in bronze ones, while the diglucosides (quercetin-3,4′-*O*-diglucoside) were only present in red cultivars, consistently in China and USA. Interestingly, the ratio of mono-/di- glucoside flavonols varied among the muscadine grapes growing in different regions, for example, the ratio ranged from 2.3 in TA-FL growing “Noble” to 7.9 in NN-GX growing “Alachua”. Furthermore, a higher content of acylated form was identified in “Noble” grape, which was likely benefited from a richer content of dihydroquercetin caffeoyl glucoside.

**Figure 2:**
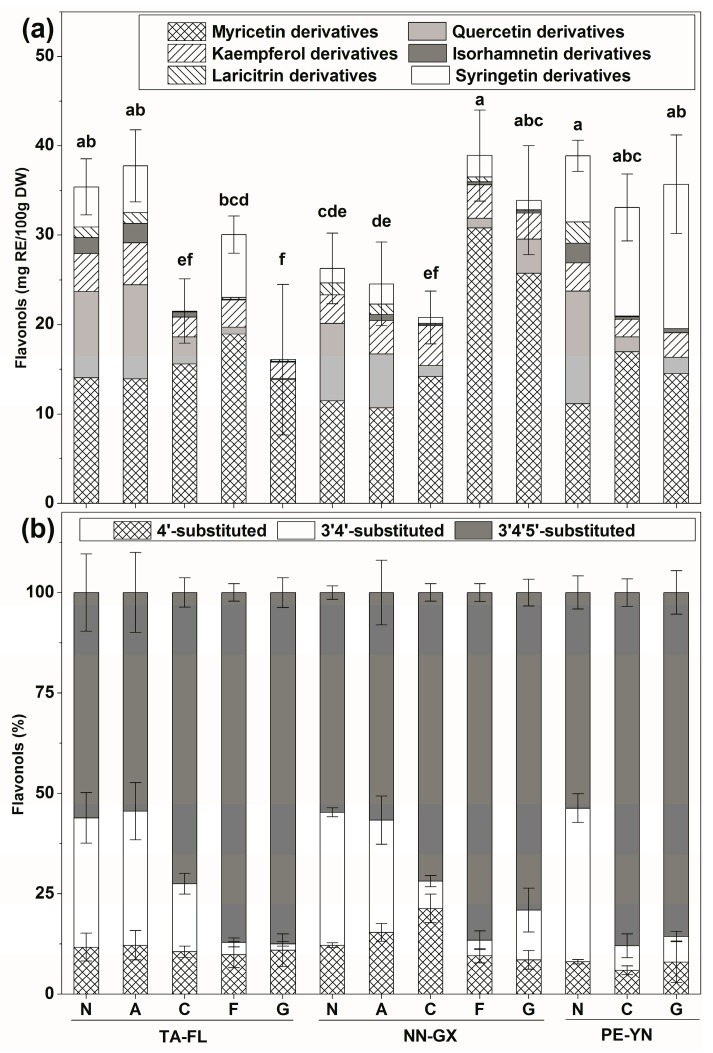
Flavonols derivatives (**a**) and substituted flavonols (**b**) distribution in five muscadine grape skins among three different regions in the 2012 season. On the top of each column, standard deviation is shown for the total content, and different small letters indicate significant differences (Duncan’s test, *p* = 0.05). See Table 1 for the abbreviation of cultivars and regions.

Myricetin derivative was the dominant type among the skin flavonol derivatives, followed by quercetin, syringetin and kaempferol derivatives, which differed among cultivars and growing regions. Myricetin derivative was significantly higher in cv. “Fry” and “Granny Val” from NN-GX (Figure 2a), while the quercetin derivative was higher in “Noble” grape. Interestingly, kaempferol derivative was mainly observed in red cultivars from TA-FL, whereas the syringetin derivative was significantly accumulated in bronze cultivars from PE-YN. Quercetin, isorhamnetin and kaempferol derivatives were the main flavonols in the muscadine seeds.

The 3′,4′,5′-substituted flavonol (myricetin, laricitrin, and syringetin derivatives) content was the highest in skins (Figure 2b). This substituted flavonol was significantly higher in bronze cultivars than the red ones. However, the 3′,4′-substituted (quercetin and isorhamnetin derivatives) was the second most common flavonol, particularly in red cultivars. In addition, 3′,4′-substituted flavonol was the main one in seeds, followed by 4′-substituted flavonol (kaempferol derivatives).

#### 2.2.3. Benzoic Acid Profiles

There were great differences of benzoic acids contents among the muscadine grapes growing in the three regions (Table 2). In the 2012 season, the grapes from NN-GX showed the highest levels of skin benzoic acids, especially for “Noble” cultivar that had 36.60 mg GAE/100 g DW. This result may be due to the higher brevifolin carboxylic acid content in the muscadine skins from NN-GX (Table S2). The seeds possessed significantly lower content of benzoic acids in comparison to the skins. In addition, both di- and mono- hydroxy benzoic acid were detected among the muscadine grapes investigated. There was higher ratio of di-/mono- hydroxy benzoic acid in the skins than in the seeds.

#### 2.2.4. Flavan-3-ols Profiles

A small amount of flavan-3-ols was detected in muscadine skins (<0.1 mg (−)-epicatechin equivalent (EE)/100 g DW), while flavan-3-ols was the second highest polyphenol found in the seeds (> 89 mg EE/100 g DW) (Table 2). The seed flavan-3-ols levels also varied significantly among cultivars grown in China and the USA, with the exception of “Alachua”, which were consistent among all the regions studied in the 2012 season. Overall, with a few exceptions, the bronze cultivars possessed higher contents of flavan-3-ols than the red ones. For instance, the “Granny Val” in PE-YN had 312.45 mg EE/100 g DW of seed flavan-3-ols.

**Figure 3:**
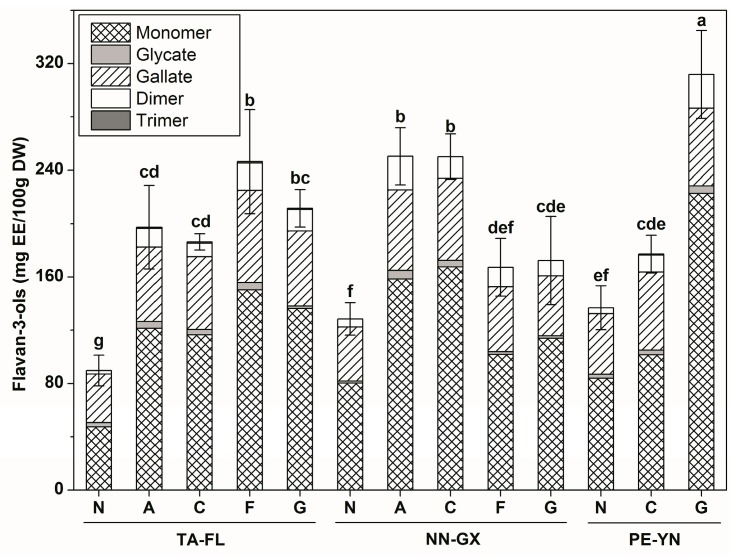
Flavan-3-ols distribution in five muscadine seeds among three different regions in the 2012 season. On the top of each column, standard deviation is show for the total content, and different small letters indicate significant differences (Duncan’s test, *p* = 0.05). See Table 1 for the abbreviation of cultivars and regions.

For flavan-3-ols constitutes, seven flavan-3-ols, mostly in their monomer forms (Tables S1–S3), were found in the skin samples. On the other hand, there were eleven flavan-3-ols detected in muscadine seeds, including two monomers, three gallates, one hexoside, three dimers, and two trimers. Monomers and gallates were the major types in all of the tested samples, accounting for 52.44%–71.27% and 18.66%–41.15% of total flavan-3-ols, respectively (Figure 3). Furthermore, the highest and lowest monomers were observed in cultivar “Granny Val” from PE-YN and “Noble” from TA-FL, respectively, whereas cultivars “Noble” and “Fry” from TA-FL showed extremely high and low levels of gallates, respectively.

#### 2.2.5. Stilbenes and Cinnamon Acids Profiles

The stilbene contents in the five muscadine skins ranging from 0 to 0.24 mg resveratrol equivalent (REE)/100 g DW among different samples, were considerably low in this study (Table 2). The ‘Carlos’ from NN-GX had the highest levels of skin stilbenes (0.18 and 0.24 mg REE/100 g DW in 2012 and 2013 season, respectively) than others. With the two individual stilbenes, resveratrol was commonly found in all the cultivars, whereas resveratrol-3-*O*-glucoside was only detected in “Carlos” from NN-GX (Table S2). A small amount of cinnamon acids was only discovered for bronze cultivars in 2012 season. Nevertheless, the stilbenes and cinnamic acids were not identified in seeds in our study.

### 2.3. Principal Component Analysis (PCA)

**Figure 4:**
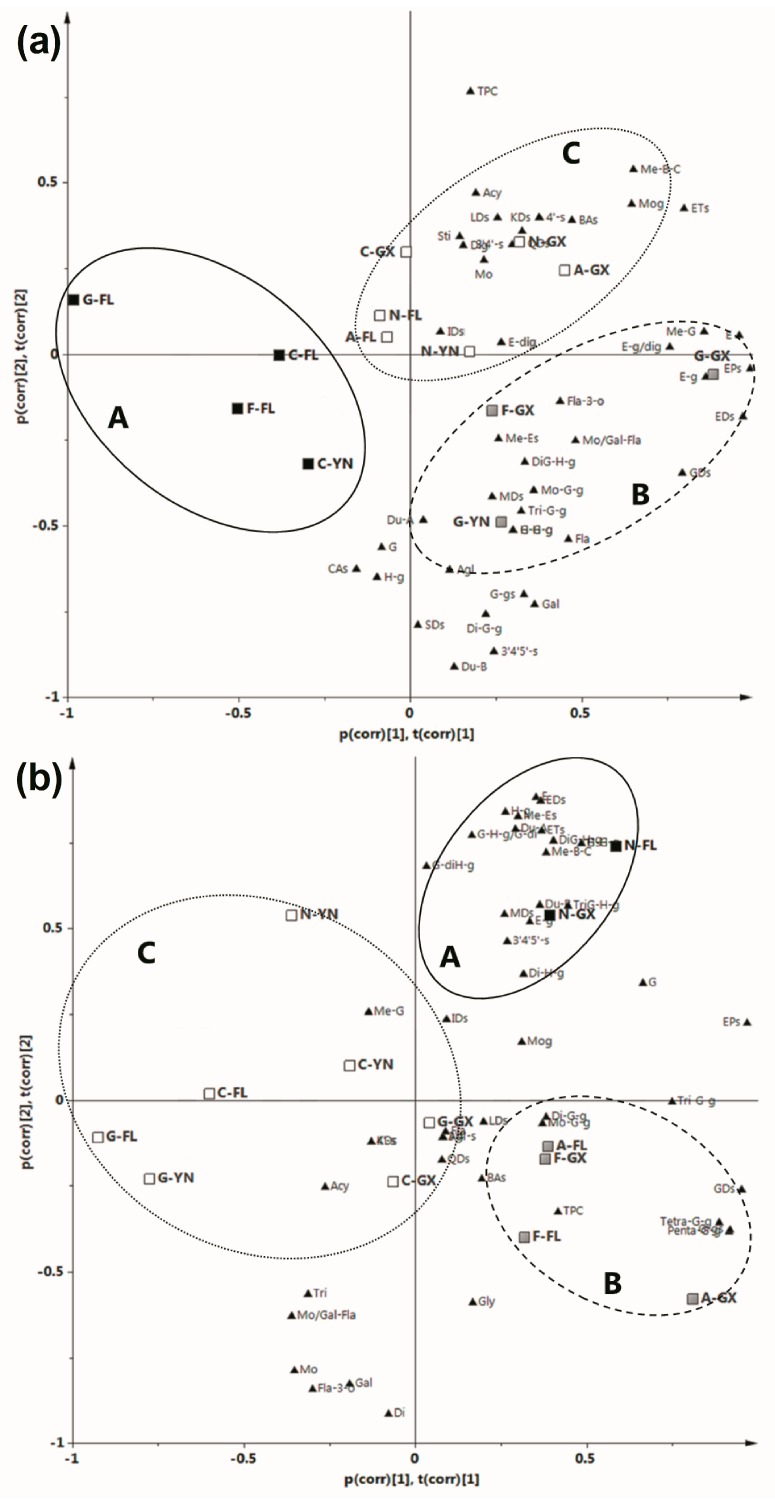
PCA biplot of loadings plot (triangle, polyphenol compounds, see Table 3) and scores plot (box, cultivars and regions) of five muscadine grape skins (**a**) and seeds (**b**). The dates were pareto scaled and the groups were classed by HCA through Ward's method. N (A, C, F, G)-FL (GX, YN): cv. “Noble” (“Alachua”, “Carlos”, “Fry”, “Granny Val”) from Tallahassee- Florida (Nanning-Guangxi, Pu’er-Yunnan).

Fifty-four evaluation parameters (Table 3) in all the samples studied were subjected to principal component analysis (PCA), in order to separate grapes according to their polyphenol characteristics. In the skins, the first three principal components (PCs) explained 83.7% of total variance (56.4%, 14.4%, and 12.9% for PC1, PC2, and PC3, respectively), which indicated that these factors were sufficient for further discussion. Figure 4a shows the skin distribution of the PCA biplot (loadings plot combined with scores plot) in two-dimensional space for PC1 and PC2. In this figure it is possible to note that the skin samples revealed three distinct groups. Group A, located in quadrants II and III, included three bronze cultivars from TA-FL and cultivar ‘Carlos’ from PE-YN. This group was matched through low values of total ellagic acids and precursors, total ellagic acid derivatives, total ellagitannins, ellagic acid, and ellagic acid glucoside/diglucoside (Table 3). Group B, in quadrant IV, was comprised of cultivar “Fry” from NN-GX, and “Granny Val” from NN-GX and PE-YN. This group was correlated with high total ellagic acids and precursors, total ellagic acid derivatives, ellagic acid, ellagic acid glucoside/diglucoside, ducheside B, total flavonols, myricetin derivatives, and 3′,4′,5′-substituted flavonols, and low TPC. Red cultivars from all the three regions and “Carlos” form NN-GX comprised group C, located in quadrants I and II. This group was characterized by the abundant TPC, total ellagitannins, and quercetin derivatives, as well as the lower ducheside B and 3′,4′,5′-substituted flavonols.

For the seeds, the first two PCs described 83.8% of total variance (55.2% for PC1, and 28.6% for PC2). The PCA biplots of seed samples also were classified into three distinct groups (Figure 4b). Group A, consisting of cultivar “Noble” from TA-FL and NN-GX, located in quadrant I, profited from high values of total ellagic acids and precursors, total ellagic acid derivatives, total ellagitannins, ellagic acid, sum of three methyl-ellagic acids, ducheside B, and low levels of total flavan-3-ols, monomer and gallate of flavan-3-ols (Table 3). Group B, in quadrant IV, was comprised of cultivars “Alachua” and “Fry” from TA-FL and NN-GX. This group was contributed mostly not only by high contents of total ellagic acids and precursors, total gallic acid derivatives, sum of five galloyl-glucoses, and penta-*O*-galloyl-glucose, and also by low total ellagic acid derivatives, and ellagic acid. Others comprised group C, located in quadrants II, III, and IV, and were linked by low levels of total ellagic acids and precursors, total gallic acid derivatives, sum of five galloyl-glucoses, and penta-*O*-galloyl-glucose.

## 3. Discussion

The chemical diversity of grapes is mostly affected by secondary metabolites represented by different phytochemical groups such as polyphenols, terpenoids, and tannins, among others, which have been attracted research interest owing to their biological activity [26]. As expected, polyphenol synthesis and accumulation in grapes is influenced by multiple factors, for instance, genotype/cultivar, geographic origin, and environmental conditions [27]. Among them, genotype plays a pivotal role in the polyphenol contents of grapes [5,7,12,28]. In this study, “Alachua” possessed the highest TPC among the muscadine cultivars investigated, which is in agreement with the findings of Sandhu and Gu [13].

Grape polyphenol compounds are greatly affected by environmental conditions such as temperature, solar radiation, sunshine duration, and rainfall [11,21]. However, a quick search of the literature demonstrates that only a few reports have addressed the effects of these parameters on TPC, ellagic acids and precursors concentration in muscadine grapes. Seasonal changes in temperature and day length are considered to be the main factors influencing the content of ellagic acids [29]. For example, there were negative correlations of overall average temperature with TPC, total tannin, and punicalagin concentration in pomegranate, which means the lower average temperature during maturing and harvest periods could promote primary polyphenol accumulation [30]. The day and night temperatures as well as the difference between them were found to play an important role in polyphenol accumulation of *Vitis* grapes [11]. Wang and Camp [29] investigated the influence of day/night temperature combinations after blooming on strawberry growth and fruit quality, and found the fruit grown at 18/12 °C contained greater amounts of ellagic acid. As the day/night temperature increased, fruit ellagic acid content decreased, while the anthocyanin content increased. This finding showed that the decrease in ellagic acid at high temperature may be due to the inhibition of ellagic acid biosynthesis or enhancement of degradation. In this sense, the average temperature and the difference between day and night in TA-FL of the USA (33.4/22.2 °C, range of temperature 11.2 °C) was larger than that in PE-YN (26.3/19.4 °C, range of temperature 6.9 °C) and NN-GX (32.9/25.4 °C, range of temperature 7.5 °C) of China before 30 days of harvest, which might lead to lower total and individual ellagic acids contents in muscadine grape skins from TA-FL.

Solar radiation and sunshine duration influence grape polyphenol synthesis and accumulation [11]. The relatively long illumination time could accumulate higher polyphenol in grapes [23,31]. For example, grapes exposed to increased daylight are capable of increased flavan-3-ol biosynthesis [32]. However, the stronger sunlight intensity results in higher berry temperature, which could cause a decrease of polyphenol in the berry [11,33]. Based on this concern, in the three regions, PE-YN (22.82° N) and NN-GX (22.47° N) was in the south of TA-FL (30.43° N), in addition, the sunshine duration for the Hengduan Mountains Region of PE-YN in the southwest of China was longer (average annual sunshine 2038.4 h), contrasting with other two plain regions of NN-GX and TA-FL (1585 and 1883 h, respectively), therefore, the former two had abundant solar radiation and sunshine duration. These differentiations should be considered as an important factor leading to increase photosynthesis and result in higher seed flavan-3-ol contents in PE-YN.

Water availability is another climatic factor affecting the synthesis and accumulation of polyphenol in grape berries. Appropriate vine water stress could lead to high anthocyanin and tannin contents [34]. One possible explanation for these results is the enhancement of TPC in water-stressed berries triggered by a reduced berry size and weight, followed by a higher proportion of fruit achene to flesh [35]. Another reason is that under these conditions, carbon should be preferentially allocated to the synthesis of primary metabolites, the level of which are not detrimental but promote the synthesis of carbon-based secondary metabolites, primarily polyphenols [30]. In addition, water deficits also increased the expression of many genes responsible for the biosynthesis of trihydroxylated anthocyanins in grape berries [36]. In our study, the average monthly rainfall before 30 days of harvest in the USA (TA-FL) was 184.9 mm (average of 1981–2010), which was lower than in China (218.8 mm in NN-GX, and 324.3 mm in PE-YN), as was the content of accumulated abundant upstream trihydroxylated anthocyanin compounds such as 3′,4′,5′-droxylated flavonols in TA-FL seeds. It is noteworthy that less than 100 mm of rainfall between veraison and maturing is an important indicator for the selection of superior wine-producing areas in *V. vinifera* [11], whereas the muscadine grapes could exhibit dominant polyphenol with the rainfall over 180 mm. This appeared to reflect better potentialities for adapting to humid climates for muscadine grapes.

In general, less is known about the impact of altitude factor on phytochemical composition. Doumet et al. [37] considered no significant differences were detected with respect to polyphenol contents and radical scavenging activity, in *Fragaria vesca* grown under the same environmental conditions but different altitude. Nevertheless, Guerrero–Chavez et al. [38] found anthocyanin concentration correlated negatively with the increase of altitude, and the strawberry antioxidant potential, measured by flow injection analysis with amperometric detection, was lower in fruits grown at higher altitude (900 vs. 1500 m). In our study, PE-YN had the highest flavonols of syringetin derivatives, which might due to the higher altitude (>1300 m) in comparison to NN-GX and TA-FL (<80 m).

However, the variation in metabolite content of ellagic acids is often correlated with data derived from genetics, transcriptomics, and environmental factors. Based on PCA and hierarchical cluster analysis (HCA), various cultivars grown in different locations could be grouped together and vice versa. The same cultivars could fall in different groups when they were cultivated in different regions. This is the result of interaction between genotype and environmental conditions which apparently influence the polyphenol synthesis and accumulation. More research is needed and multiple factors must be coordinately considered at the same time, in order to better understand the role played by the independent and/or mutual influence factors of ellagic acids biosynthesis in muscadine grapes, especially when global warming is intensifying, which affects many viticulture areas in the world.

## 4. Materials and Methods

### 4.1. Chemicals

All chemicals and standards mentioned below were of HPLC grade. Acetonitrile and methanol were purchased from Thermo Fisher Scientific (Waltham, MA, USA). Formic acid was supplied from VWR (Helsinki, Finland). Standard caffeic acid (≥98%), ellagic acid (≥98%), (−)-epicatechin (≥90%), gallic acid (≥98%), kaempferol (≥97%), myricetin (≥96%), quercetin (≥98%), and rutin (≥95%) were obtained from Sigma-Aldrich (St. Louis, MO, USA). Penta-*O*-galloyl-glucose (≥99%) and resveratrol (≥98%) were purchased from Solarbio (Beijing, China). Ultra-pure water from a Millipore Synergy water purification system (Merck KGaA, Darmstadt, Germany) with conductivity of 18 MΩ was used throughout. Other reagents were analytical pure and obtained from Beijing Chemical Works (Beijing, China).

### 4.2. Grape Materials

Fruits of five fully ripened muscadine grape cultivars (red cv. “Noble” and “Alachua”, and bronze cv. ”Carlos”, “Fry”, and “Granny Val”) were collected from Tallahassee-Florida, United States (TA-FL) and Nanning-Guangxi, China (NN-GX), likewise, grapes of three cultivars (cv. “Noble”, “Carlos”, and “Granny Val”) were obtained from Pu’er-Yunnan, China (PE-YN), for two consecutive years (2012 and 2013). Grapes from three to four clusters per vine, and ten vines per target cultivar were picked randomly throughout the vineyard, taking into account the balance between shadow and sun exposure, and following a z-shaped pattern to avoid edge and center effects [26]. Samples were transported immediately under refrigeration (ca. 2–5 °C) to the laboratory. Skin and seed fractions were separated manually, freeze-dried (LGJ-18, Songyuan Huaxing Biotechnology Co., LTD, Beijing, China) 48 h, and stored at −80 °C.

**Figure 5:**
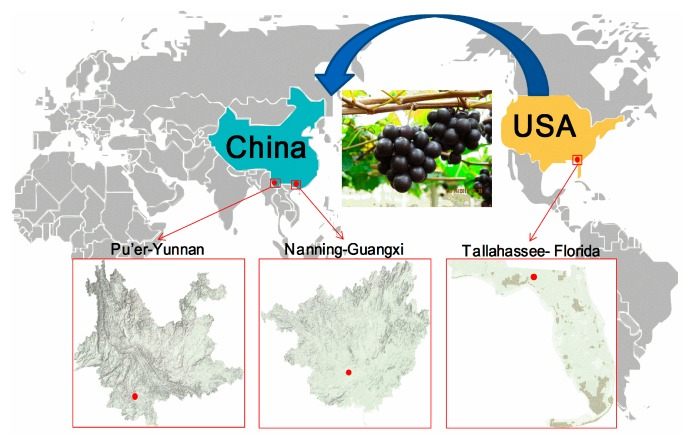
The three region distribution of muscadine grapes used in this study.

To illustrate the regional difference of the three sampling locations, consider the geographical distribution presented in Figure 5. Based on the data from the America’s National Oceanic and Atmospheric Administration and China Meteorological National Administration (1981–2010), all these regions have a warm and humid subtropical climate, with long summers and short, mild winters. TA-FL is located at 30.43° N latitude, 84.26° W longitude, and has an altitude of 62 m. The annual average temperature is 19.8 °C (27.8 °C in July, and 10.7 °C in January), the annual rainfall is 1506 mm, and the sunshine lasts 1883 h. NN-GX is located at 22.47° N latitude, 108.21° E longitude, with an altitude of 80 m. The annual average temperature is 21.8 °C (28.4 °C in July, and 12.9 °C in January), the annual rainfall is 1310 mm, and the sunshine lasts 1585 h. PE-YN is located at 22.82° N latitude, 100.97° E longitude, and has an altitude above 1306 m. The annual average temperature is 19.5 °C (23.2 °C in July, and 13.7 °C in January), the annual rainfall is 1497 mm, and the city receives 2038 hours of bright sunshine annually.

### 4.3. Extraction and Determination of Polyphenol in Muscadine Grapes

#### 4.3.1. Preparation of Berries Extraction

Freeze-dried grape seeds were crushed and then defatted as previously reported [11,39]. Briefly, freeze dried grape seeds were moderately crushed by a stainless-steel grinder (FW-135, Tester Co., LTD, Tianjin, China), then defatted with petroleum ether at a ratio of 1:20 (*w*/*v*). After 12 h extraction at room temperature and in the dark, the liquid was separated from the solid by vacuum filtration (T-50, Jinteng Experiment Equipment Co., LTD, Tianjin, China) through a sintered glass filter (Pyrex, porosity 10–15 µm). The defatted procedure was carried out twice and the solid residue was evenly distributed over a culture dish, then kept in dark 6 h for evaporation of petroleum ether. The ultimate defatted grape seeds were put into a mortar containing liquid nitrogen and ground into powder as fine as possible. Freeze-dried grape skins were ground with the stainless-steel grinder to pass 60 sieve sizes (0.25 mm), then both were stored at −80 °C.

#### 4.3.2. Extraction of Polyphenols in Muscadine Grapes

Polyphenol compounds were extracted from skins and seeds as previously reported in our laboratory [11,39]. Briefly, 0.5 g freeze-dried skins or defatted seeds were placed into a 50-mL centrifuge tube with methanol/water/hydrochloric acid (70:29:1, *v*/*v*/*v*) at a ratio of 1:30 (*w*/*v*), vortexed for 15 s (HMQL-VORTEX-5, Midwest Group, Beijing, China), then extracted at 616 W for 28 min in an ultrasonic cleaning machine (SB-5200, Ningbo Scientz Biotechnology Co., LTD, Ningbo, China) at 25 °C. After centrifuging at 7600 rpm for 20 min (Allegra X-30R, Beckman Coulter Inc., Brea, CA, USA), the supernatant was collected and the precipitate was re-extracted two more times. The supernatant was combined and the solvent was removed by vacuum evaporation (RE-52, Shanghai YaRong Biochemical Instrument Factory, Shanghai, China) at 35 °C. The solids obtained after evaporation were re-dissolved in 5 mL of methanol (1% formic acid) and stored at −80 °C. Extractions were performed in three replicates for all individual samples. Samples were filtered by 0.22 μm of cellulose membrane (Jinteng Experiment Equipment Co., LTD, Tianjin, China) and then detected by UPLC Triple TOF-MS/MS.

#### 4.3.3. Determination of TPC

The TPC was determined by Folin–Ciocalteu colourimetric method as previously reported in our laboratory [11,39]. Briefly, all samples were diluted. Folin–Ciocalteu reagent and sodium carbonate were successively added. The solution was reacted at 40 °C for 30 min, and then the absorbance was read at 760 nm by a UV-2800 spectrometer (UNICO, Suite E Dayton, NJ, USA). Gallic acid was used as standard and values were expressed as gallic acid equivalent dry weight (mg GAE/g DW), with the linearity range 50–1000 μg/mL (*R^2^* > 0.999).

#### 4.3.4. UPLC-Triple TOF-MS/MS Analysis

Sample analysis was carried out using an Eksigent ultraLC 110 and a Triple TOF 4600-MS/MS (AB SCIEX, Framingham, MA, USA) coupled with a Duospray Ion Source interface and automatic Calibrant Delivery System (CDS). The ultraLC 110 includes an online degasser, a double pump, an autosampler, and a thermostatic column control system, all of which were controlled by Analyst^®^ TF 1.6 software. The UPLC separation was performed on a reversed-phase Zorbax SB-C18 column (2.1 mm × 150 mm × 5 μm, Agilent Technologies, Santa Clara, CA, USA) at 35 °C. The mobile phase was water with 0.5% formic acid (A) and acetonitrile (B) at the following gradient: 0–1 min, 5% B; 1–20 min, 5%–60% B; 20–21 min, 60%–95% B; 21–30 min, column wash and stabilization. The flow rate was 0.3 mL/min and the injection volume was 10 μL. MS conditions: ion source gas 1 and 2 (air), 55 psi; curtain gas (N_2_), 30 psi; source temperature, 550 °C; ionspray voltage, −4.5 kv; collision energy, −40 ± 10 eV, scan from *m*/*z* 100 to 2000. Tuning Solution in Installation Kit and APCI Negative Calibration Solution (AB SCIEX) were used to monitor the stability of the ionization efficiency of the mass spectrometer and the *m*/*z* values of Triple TOF systems.

Data acquisition and processing was performed using Peak View 2.0 and Marker View 1.2.1 software (AB SCIEX). The polyphenol compounds were identified based on total ion chromatogram, retention time, exact molecular weight, and Triple TOF MS/MS fragmentation characteristics, such as the representative description in Figures S1–S3 and Table S4.

Multi Quant 3.0 software (AB SCIEX) was used for quantitative analysis. Caffeic acid, ellagic acid, (−)-epicatechin, gallic acid, kaempferol, myricetin, penta-*O*-galloyl-glucose, quercetin, resveratrol, and rutin were quantified by their standards, respectively, and expressed as mg/100 g DW. Other benzoic acids, cinnamic acids, ellagic acids, flavan-3-ols, flavonols, and stilbens were respectively expressed as micrograms of gallic acid equivalent (GAE), caffeic acid equivalent (CAE), GAE, (−)-epicatechin equivalent (EE), rutin equivalent (RE), and resveratrol equivalent (REE)/100 g DW. The CDS was adjusted every five hours when sample running, the standard curves were produced every day with three parallel measurements. The linear ranges of different standards were 0–5, 0–10, and 10–50 mg/L, corresponding to the concentrations (*R^2^* > 0.999).

### 4.4. Statistical Analysis

Results were expressed as means of three parallel measurements ± S.D. Microsoft Excel 2010, SPSS 20.0 (IBM, Armonk, NY, USA), and Origin 8.5 (Origin Lab, Northampton, MA, USA) software were used for data processing and graphing. Significance difference was tested by ANOVA (Duncan’s test, *p* = 0.05). Principal component analysis (PCA) and hierarchical cluster analysis (HCA) were performed by SIMCA-P 13.0 (Umetrics, Malmö, Sweden).

## 5. Conclusions

Ellagic acids and precursors were the characteristic polyphenols detected in muscadine grapes. Our research was the first study to analyze the ellagic acids and precursor composition and accumulation in muscadine grapes grown in South China (NN-GX, and PE-YN). Fourteen new ellagitannins (thirteen in skins, and one in seeds) were identified in muscadine grapes for the first time. Multiple factors influenced the polyphenol synthesis and accumulation. Differences were observed varied within and between grape genotype/cultivars (white and red) and grape fractions (skins and seeds), as well as in different regions under different environmental conditions. Based on PCA, the cultivars from different regions were classified into three distinct groups, in both skins and seeds, presenting characteristic and discriminative variances. These results indicated that muscadine grapes could be grown well in countries besides the USA.

## Figures and Tables

**Table 1:** Total phenolic content (TPC) in skins and seeds of five muscadine grapes cultivated in three different regions.

Cultivar	Region	Year	TPC (mg GAE/g DW)		
			Skins	Seeds	Skins + Seeds
**Red cultivars**					
Noble (N)	TA-FL	2012	38.96 ± 2.57 ^d^	96.65 ± 3.30 ^a^	135.61 ± 5.17 ^c^
	NN-GX	2012	42.06 ± 2.00 ^c^	82.10 ± 5.15 ^b^	124.16 ± 6.55 ^d^
		2013	48.08 ± 0.67 ^b^	95.07 ± 1.07 ^a^	143.14 ± 0.95 ^b^
	PE-YN	2012	41.40 ± 1.93 ^c^	85.44 ± 3.40 ^b^	126.84 ± 4.32 ^d^
		2013	52.68 ± 1.91 ^a^	97.55 ± 3.04 ^a^	150.23 ± 3.19 ^a^
	Sig. ^a^		**	***	***
Alachua (A)	TA-FL	2012	36.21 ± 1.70 ^b^	118.59 ± 2.51 ^a^	154.80 ± 3.33 ^b^
	NN-GX	2012	45.03 ± 1.35 ^a^	107.05 ± 3.48 ^b^	152.08 ± 4.28 ^b^
		2013	46.12 ± 2.82 ^a^	119.41 ± 4.14 ^a^	165.53 ± 1.49 ^a^
	Sig. ^a^		***	***	ns
	Sig. ^b^		**, **	***, ***	***, ***
**Bronze cultivars**					
Carlos (C)	TA-FL	2012	30.86 ± 4.88 ^c^	103.74 ± 4.50 ^b^	134.60 ± 7.92 ^c^
	NN-GX	2012	35.98 ± 1.58 ^b^	90.32 ± 5.06 ^c^	126.30 ± 5.38 ^d^
		2013	56.44 ± 1.37 ^a^	122.55 ± 1.61 ^a^	178.99 ± 1.96 ^a^
	PE-YN	2012	30.22 ± 1.50 ^c^	83.83 ± 4.80 ^d^	114.05 ± 4.94 ^e^
		2013	57.33 ± 2.07 ^a^	102.87 ± 3.01 ^b^	160.20 ± 3.58 ^b^
	Sig. ^a^		***	***	***
Fry (F)	TA-FL	2012	27.43 ± 4.25 ^b^	118.74 ± 3.47 ^a^	146.17 ± 6.69 ^b^
	NN-GX	2012	38.68 ± 1.83 ^a^	77.88 ± 3.81 ^c^	116.56 ± 3.54 ^c^
		2013	39.80 ± 2.02 ^a^	113.49 ± 2.78 ^b^	153.29 ± 3.70 ^a^
	Sig. ^a^		***	***	***
Granny Val (G)	TA-FL	2012	34.44 ± 2.02 ^b^	76.56 ± 2.28 ^d^	111.00 ± 3.83 ^c^
	NN-GX	2012	29.34 ± 2.41 ^c^	81.14 ± 1.73 ^c^	110.48 ± 3.21 ^c^
		2013	44.92 ± 1.08 ^a^	116.36 ± 1.21 ^a^	161.28 ± 0.94 ^a^
	PE-YN	2012	20.53 ± 0.78 ^d^	85.78 ± 2.94 ^b^	106.31 ± 3.24 ^d^
		2013	35.38 ± 1.08 ^b^	114.13 ± 3.46 ^a^	149.51 ± 3.85 ^b^
	Sig. ^a^		***	***	*
	Sig. ^b^		***, ***	***, ***	***, ***

Values are expressed as means of triplicate determinations ± standard deviation S.D. For each cultivar, different small letters within column indicate significant differences (Duncan’s test, *p* = 0.05). Significance among different regions for each cultivar in the 2012 season (Sig. ^a^), and among different color cultivars grown in TA-FL and NN-GX in 2012 (Sig. ^b^) was tested for *p* < 0.001 (***), *p* < 0.01 (**), *p* < 0.05 (*), and not significant (ns), respectively. TA-FL: Tallahassee-Florida; NN-GX: Nanning, Guangxi; PE-YN: Pu’er-Yunnan; GAE: gallic acid equivalent.

**Table 2:** Different categories of polyphenol contents in skins and seeds of five muscadine grapes cultivated in three different regions.

Cultivar	Region	Year	Ellagic Acids and Precursors (mg GAE/100 g DW)		Flavonols (mg RE/100 g DW)		Benzoic Acids (mg GAE/100 g DW)		Flavan-3-ols (mg EE/100 g DW)		Stilbenes (mg REE/100 g DW)		Cinnamic Acids (mg CAE/100 g DW)	
			Skins	Seeds	Skins	Seeds	Skins	Seeds	Skins	Seeds	Skins	Seeds	Skins	Seeds
**Red cultivars**														
Noble (N)	TA-FL	2012	174.30 ^b^	636.75 ^a^	35.42 ^c^	18.96 ^a^	19.49 ^b^	4.20 ^d^	nd	89.72 ^c^	0.06 ^b^	nd	nd	nd
	NN-GX	2012	197.25 ^b^	557.76 ^b^	26.28 ^d^	7.03 ^c^	36.60 ^a^	19.10 ^b^	0.08 ^a^	128.43 ^b^	nd	nd	nd	nd
		2013	255.66 ^a^	670.73 ^a^	57.52 ^b^	13.03 ^b^	39.00 ^a^	34.90 ^a^	nd	216.25 ^a^	nd	nd	nd	nd
	PE-YN	2012	196.85 ^b^	380.61 ^d^	38.89 ^c^	10.48 ^b,c^	18.00 ^b^	7.18 ^c,d^	0.05 ^b^	136.88 ^b^	0.06 ^b^	nd	nd	nd
		2013	299.12 ^a^	468.22 ^c^	66.00 ^a^	14.76 ^a,b^	40.36 ^a^	10.96 ^c^	nd	230.45 ^a^	0.11 ^a^	nd	nd	nd
	Sig. ^a^		ns	***	**	***	***	**	n/a	*	n/a	n/a	n/a	n/a
Alachua (A)	TA-FL	2012	174.31 ^c^	510.76 ^b^	37.74 ^b^	21.26 ^a^	17.04 ^a^	6.75 ^b^	0.02 ^b^	197.61 ^b^	nd	nd	nd	nd
	NN-GX	2012	220.99 ^b^	577.89 ^a^	24.54 ^c^	9.50 ^b^	7.51 ^b^	19.11 ^a^	0.05 ^a^	250.96 ^b^	0.06 ^a^	nd	nd	nd
		2013	294.57 ^a^	512.46 ^b^	55.20 ^a^	28.85 ^a^	9.72 ^b^	0.36 ^c^	0.03 ^a,b^	369.42 ^a^	0.06 ^a^	nd	nd	nd
	Sig. ^a^		*	ns	*	*	*	**	**	ns	n/a	n/a	n/a	n/a
	Sig. ^b^		ns, **	*, ns	ns, ns	ns, *	ns, ***	*, ns	***, **	**, ***	n/a, n/a	n/a, n/a	n/a, n/a	n/a, n/a
**Bronze cultivars**														
Carlos (C)	TA-FL	2012	144.94 ^d^	257.21 ^c^	21.52 ^c^	20.69 ^c^	12.28 ^b,c^	4.93 ^b,c^	0.05 ^b^	186.56 ^b^	nd	nd	nd	nd
	NN-GX	2012	169.89 ^c^	406.42 ^b^	20.79 ^c^	10.52 ^d^	21.42 ^a^	7.22 ^a^	0.08 ^b^	250.65 ^a^	0.18 ^a^	nd	0.14 ^b^	nd
		2013	212.85 ^b^	457.55 ^a,b^	31.66 ^b^	41.60 ^a^	16.12 ^b^	4.34 ^c^	0.24 ^a^	266.21 ^a^	0.24 ^a^	nd	nd	nd
	PE-YN	2012	155.07 ^c,d^	381.49 ^b^	33.10 ^b^	14.67 ^c,d^	6.81 ^d^	6.14 ^a^^,^^b^	0.06 ^b^	177.49 ^b^	nd	nd	0.22 ^a^	nd
		2013	264.76 ^a^	514.50 ^a^	54.75 ^a^	29.06 ^b^	7.77 ^c^^,^^d^	1.71 ^d^	0.02 ^b^	258.52 ^a^	0.03 ^b^	nd	nd	nd
	Sig. ^a^		*	***	**	***	**	**	*	***	n/a	n/a	n/a	n/a
Fry (F)	TA-FL	2012	132.30 ^c^	482.50 ^a^	30.06 ^c^	27.89 ^b^	13.31 ^b^	9.14 ^a^	0.02 ^b^	246.94 ^a^	nd	nd	0.11	nd
	NN-GX	2012	201.26 ^b^	486.00 ^a^	39.95 ^b^	7.98 ^c^	22.37 ^a^	6.62 ^b^	0.07 ^a^	167.81 ^b^	nd	nd	nd	nd
		2013	273.25 ^a^	475.86 ^a^	60.60 ^a^	38.97 ^a^	6.59 ^c^	5.50 ^b^	nd	240.49 ^a^	0.12	nd	nd	nd
	Sig. ^a^		*	ns	*	**	**	*	***	*	n/a	n/a	n/a	n/a
Granny Val (G)	TA-FL	2012	85.42 ^d^	153.14 ^d^	16.07 ^c^	10.17 ^b^	16.41 ^b,c^	4.93 ^b^	0.02 ^c,d^	212.01 ^b,c^	0.06 ^a^	nd	0.19 ^b^	nd
	NN-GX	2012	248.73 ^b^	398.08 ^b^	33.90 ^b^	9.82 ^b^	33.97 ^a^	19.65 ^a^	0.04 ^b,c^	172.55 ^c^	0.06 ^a^	nd	nd	nd
		2013	295.44 ^a^	582.68 ^a^	44.83 ^a,b^	31.56 ^a^	14.80 ^b,c^	11.66 ^a,b^	0.06 ^a,b^	250.59 ^b^	0.09 ^a^	nd	nd	nd
	PE-YN	2012	201.41 ^c^	224.19 ^c^	35.69 ^a,b^	12.13 ^b^	11.90 ^a^	18.83 ^a^	0.09 ^a^	312.45 ^a^	0.06 ^a^	nd	0.59 ^a^	nd
		2013	268.67 ^a,b^	281.51 ^c^	47.86 ^a^	23.60 ^a^	23.53 ^a,b^	10.83 ^a,b^	nd	319.56 ^a^	nd	nd	nd	nd
	Sig. ^a^		***	***	*	ns	*	ns	**	**	ns	n/a	n/a	n/a
	Sig. ^b^		*, **	***, ns	ns, **	***, ns	ns, ns	***, ns	***, **	ns, *	n/a, n/a	n/a, n/a	n/a, n/a	n/a, n/a

Values are expressed as means of triplicate determinations. For each cultivar, different small letters within column indicate significant differences (Duncan’s test, *p* = 0.05). Significance among different regions for each cultivar in the 2012 season (Sig. ^a^), and among different color cultivars grown in TA-FL and NN-GX in 2012 (Sig. ^b^) was tested for *p* < 0.001 (***), *p* < 0.01 (**), *p* < 0.05 (*), not significant (ns), and not applicable (n/a), respectively. nd: not detected; CAE: caffeic acid equivalent; EE: (−)-epicatechin equivalent; RE: rutin equivalent; REE: resveratrol equivalent. See Table 1 for the abbreviation of regions.

**Table 3:** The contribution scores of 54 evaluation parameters from different groups of muscadine skins and seeds based on principal components analysis (PCA) and hierarchical cluster analysis (HCA) in the 2012 season.

No.	Compounds	Abbreviation	Skins			Seeds		
			Group A	Group B	Group C	Group A	Group B	Group C
1	Total phenolic content	TPC	−0.4043	−**1.2307**	**1.4201**	−0.1302	0.4540	−0.2132
2	Ellagic acids and precursors	EPs	−4.0271	2.4873	0.3189	2.5816	1.8111	−2.6843
3	Ellagic acid derivatives	EDs	−3.0158	2.5967	0.1427	5.5202	−1.1154	−0.2678
4	Ellagic acid	E	−**2.7306**	**1.4526**	0.2713	**4.8190**	−**1.0040**	−0.2151
5	Methyl-ellagic acids (1–3)	Me-Es	−0.1053	0.5372	−0.2334	**1.3958**	−0.2875	−0.0571
6	Ducheside A	Du-A	−0.0512	0.1191	0.0386	0.9210	−0.2086	−0.0345
7	Ducheside B	Du-B	0.1316	**1.5802**	−**1.0522**	**1.0886**	−0.1932	−0.0910
8	Ellagic acid glucoside	E-g	−0.8667	0.8782	−0.0100	0.3727	−0.0648	−0.0317
9	Ellagic acid-dihexoside	E-dig	−0.0232	0.0385	−0.0194	−	−	−
10	Ellagic acid glu/diglu	E-g/dig	−**1.6530**	**1.4763**	0.0737	−	−	−
11	Ellagitannins	ETs	−**1.9865**	0.2552	**1.1640**	**1.6039**	−0.2092	−0.1179
12	Methyl brevifolin carboxylate	Me-B-C	−0.9633	0.4421	0.6616	0.8561	−0.0601	−0.0858
13	HHDP-glucose	H-g	0.0546	0.0342	−0.1208	0.7131	−0.1203	−0.0299
14	Pedunculagin α/β isomer (Di-HHDP-glucose)	Di-H-g	−	−	−	0.1157	−0.0191	−0.0138
15	Mono-/di-HHDP-glucose	Mo/di-H-g	−	−	−	−	−	−
16	Galloyl-HHDP-glucose (Corilagin, Strictinin)	G-H-g	−0.0325	0.1229	−0.0254	0.4223	−0.0227	−0.0541
17	Galloyl-bis-HHDP-glucose (Casuarinin)	G-diH-g	−	−	−	0.2189	−0.0866	0.00002
18	Galloyl-HHDP-glucose/Galloyl-bis-HHDP-glucose	G-H-g/G-diH-g	−	−	−	0.4264	−0.1078	−0.0077
19	HHDP-galloyl-glucose (Isostrictinin)	H-G-g	−0.0325	0.1229	−0.0254	0.4223	−0.0227	−0.0541
20	Tellimagrain I (Digalloyl-HHDP-glucose)	DiG-H-g	−0.0307	0.0334	0.0139	0.8165	−0.0951	−0.0365
21	Tellimagrain II (Trigalloyl-HHDP-glucose)	TriG-H-g	−	−	−	0.1536	0.0236	−0.0365
22	Precursors (Gallic acid derivatives)	GDs	−0.4620	0.9006	−0.1423	0.5890	**2.7441**	−**2.3172**
23	Gallic acid	G	0.0934	0.0532	−0.1932	0.5468	0.3028	−0.3943
24	Methyl gallate	Me-G	−0.6455	0.6317	0.0035	−0.1558	−0.0158	0.0232
25	Mono-*O*-galloyl-glucose	Mo-G-g	−0.0028	0.0315	−0.0143	0.0113	0.0393	−0.0362
26	Di-*O*-galloyl-glucose	Di-G-g	0.0170	0.2667	−0.1653	−0.0016	0.0059	−0.0037
27	Tri-*O*-galloyl-glucose	Tri-G-g	0.0035	0.0996	−0.0766	0.1246	0.0980	−0.1513
28	Tetra-*O*-galloyl-glucose	Tetra-G-g	−	−	−	0.1164	0.5409	−0.4237
29	Penta-*O*-galloyl-glucose	Penta-G-g	−	−	−	0.5180	**2.7015**	−**2.0699**
30	Galloyl-glucoses (1–5)	G-gs	0.0137	0.2838	−0.1641	0.5533	**2.7601**	−**2.1336**
31	Flavonols	Fla	−0.5623	**1.0174**	0.0858	−0.0143	0.0376	−0.0142
32	Myricetin derivatives	MDs	0.0022	**1.0660**	−0.7312	0.2699	−0.0538	−0.0153
33	Quercetin derivatives	QDs	−0.3407	−0.3765	**1.1126**	−0.0641	0.0734	−0.0118
34	Kaempferol derivatives	KDs	−0.2456	−0.0330	0.2954	−0.0013	−0.0213	0.0107
35	Isorhamnetin derivatives	IDs	−0.0266	−0.0245	0.3417	0.0296	0.0184	−0.0067
36	Laricitrin derivatives	LDs	−0.1156	−0.1750	0.4492	0.0073	−0.0007	−0.0028
37	Syringetin derivatives	SDs	0.0174	0.5389	−0.3337	−	−	−
38	4'-substituted flavonol	4'-s	−0.2456	−0.0330	0.2954	−0.0013	−0.0213	0.0107
39	3'4'-substituted flavonol	3'4'-s	−0.3238	−0.3563	**1.1856**	−0.0317	0.0497	−0.0140
40	3'4'5'-substituted flavonol	3'4'5'-s	−0.0401	**2.3605**	−**1.1655**	0.2033	−0.0183	−0.0211
41	Flavonol aglycone	Agl	−0.0720	0.2056	0.0156	−0.0351	0.0521	−0.0141
42	Flavonol monoglucoside	Mog	−0.6273	−0.1335	0.7024	0.1012	−0.0196	−0.0228
43	Flavonol diglucoside	Dig	−0.1058	−0.2083	0.6461	−	−	−
44	Flavonol acylation	Acy	−0.2102	−0.4368	0.9267	−0.0109	−0.0100	0.0072
45	Benzoic acids	BAs	−0.5820	0.5540	0.2280	0.0510	0.0038	−0.0110
46	Flavan-3-ols	Fla-3-o	−0.0175	0.0228	−0.0020	−**3.6329**	0.7254	0.1494
47	Flavan-3-ol monomer	Mo	−0.0021	−0.0014	0.0037	−**2.7396**	0.3694	0.1878
48	Flavan-3-ol glycate	Gly	−	−	−	−0.2402	0.1468	−0.0084
49	Flavan-3-ol gallate	Gal	−0.0077	0.1030	−0.0425	−**1.5488**	0.4488	0.0222
50	Flavan-3-ol dimer	Di	−	−	−	−0.4692	0.1855	−0.00004
51	Flavan-3-ol trimer	Tri	−	−	−	−0.2596	0.0910	0.0051
52	Flavan-3-ol monomer/gallate	Mo/Gal	−0.0103	0.1002	−0.0349	−0.2456	0.0016	0.0313
53	Stilbenes	Sti	−0.0164	−0.0003	0.0271	−	−	−
54	Cinnamic acids	CAs	0.0181	0.1248	−0.0987	−	−	−

The score contribution is described as group-average weight of first three principal components (PCs) for skins and first two PCs for seeds. −: no contribution. In bold, compounds with absolute value of contribution score >1.

## Supplementary Materials

Supplementary materials can be found at www.mdpi.com/1422-0067/18/3/631/s1.

## References

[1] Pastrana-Bonilla E., Akoh C.C., Sellappan S., Krewer G. (2003). Phenolic content and antioxidant capacity of muscadine grapes. J. Agric. Food Chem..

[2] Conner P.J. Characteristics of promising muscadine grape (*Vitis rotundifolia* Michx.) selections from the University of Georgia (USA) Breeding Program. Proceedings of the X International Conference on Grapevine Breeding and Genetics.

[3] Talcott S.T., Lee J. (2002). Ellagic acid and flavonoid antioxidant content of muscadine wine and juice. J. Agric. Food Chem..

[4] Louime C., Lu J., Onokpise O., Vasanthaiah H.K.N., Kambiranda D., Basha S.M., Yun H.K. (2011). Resistance to *Elsinoe Ampelina* and expression of related resistant genes in *Vitis Rotundifolia* Michx. grapes. Int. J. Mol. Sci..

[5] Marshall D.A., Stringer S.J., Spiers J.D. (2012). Stilbene, ellagic acid, flavonol, and phenolic content of muscadine grape (*Vitis rotundifolia* Michx.) cultivars. Pharm. Crops.

[6] Yu Y., Wu J., Fu S., Yin L., Zhang Y., Lu J. (2016). Callose synthase family genes involved in the grapevine defense response to downy mildew disease. Phytopathology.

[7] Chen W.W. (2011). Antimicrobial and Antioxidant Activity of Muscadine (*Vitis rotundifolia* Michx.) Extracts as Influenced by Solvent Extraction Methods and Cultivars. Master’s Thesis.

[8] Xu C., Yagiz Y., Hsu W., Simonne A., Lu J., Marshall M.R. (2014). Antioxidant, antibacterial, and antibiofilm properties of polyphenols from muscadine grape (*Vitis rotundifolia* Michx.) pomace against selected foodborne pathogens. J. Agric. Food Chem..

[9] Xu C., Yavuz Y., Marshall S., Li Z., Simonne A.H., Lu J., Marshall M.R. (2015). Application of muscadine grape (*Vitis rotundifolia*) pomace extract to reduce carcinogenic acrylamide. Food Chem..

[10] You Q. (2012). Biological Properties Evaluation and Chemical Profiles of Phenolic Compounds in Muscadine Grapes (*Vitis rotundifolia*). Ph.D. Thesis.

[11] Xu C., Zhang Y., Zhu L., Huang Y., Lu J. (2011). Influence of growing season on phenolic compounds and antioxidant properties of grape berries from vines grown in subtropical climate. J. Agric. Food Chem..

[12] Zhu L., Zhang Y., Lu J. (2012). Phenolic contents and compositions in skins of red wine grape cultivars among various genetic backgrounds and originations. Int. J. Mol. Sci..

[13] Sandhu A.K., Gu L. (2010). Antioxidant capacity, phenolic content, and profiling of phenolic compounds in the seeds, skin, and pulp of *Vitis rotundifolia* (Muscadine Grapes) as determined by HPLC-DAD-ESI-MS(n). J. Agric. Food Chem..

[14] Lee J., Johnson J.V., Talcott S.T. (2005). Identification of ellagic acid conjugates and other polyphenolics in muscadine grapes by HPLC-ESI-MS. J. Agric. Food Chem..

[15] Lorrain B., Chira K., Teissedre P. (2011). Phenolic composition of Merlot and Cabernet-Sauvignon grapes from Bordeaux vineyard for the 2009-vintage: Comparison to 2006, 2007 and 2008 vintages. Food Chem..

[16] Narduzzi L., Stanstrup J., Mattivi F. (2011). Comparing wild American grapes with *Vitis vinifera*: A metabolomics study of grape composition. J. Agric. Food Chem..

[17] Marshall-Shaw D.A., Stringer S.J., Sampson B.J., Spiers J.D. (2014). Storage retention of stilbene, ellagic acid, flavonol, and phenolic content of muscadine grape (*Vitis rotundifolia* Michx.) cultivars. J. Food Chem. Nutr..

[18] Hager T.J., Howard L.R., Liyanage R., Lay J.O., Prior R.L. (2008). Ellagitannin composition of blackberry as determined by HPLC-ESI-MS and MALDI-TOF-MS. J. Agric. Food Chem..

[19] Quideau S., Deffieux D., Douat-Casassus C., Pouységu L. (2011). Plant polyphenols: Chemical properties, biological activities, and synthesis. Angew. Chem. Int. Edit..

[20] García-ESstévez I., Andrés-García P., Alcalde-Eon C., Giacosa S., Rolle L., Rivas-Gonzalo J.C., Quijada-Morín N., Escribano-Bailón M.T. (2015). Relationship between agronomic parameters, phenolic composition of grape skin, and texture properties of *Vitis vinifera* L. cv. Tempranillo. J. Agric. Food Chem..

[21] Downey M.O., Dokoozlian N.K., Krstic M.P. (2006). Cultural practice and environmental impacts on the flavonoid composition of grapes and wine: A review of recent research. Am. J. Enol. Viticult..

[22] Sandhu A.K., Gray D.J., Lu J., Gu L. (2011). Effects of exogenous abscisic acid on antioxidant capacities, anthocyanins, and flavonol contents of muscadine grape (*Vitis rotundifolia*) skin. Food Chem..

[23] Lu Z., Liu Y., Zhao L., Jiang X., Li M., Wang Y., Xu Y., Gao L., Xia T. (2014). Effect of low-intensity white light mediated de-etiolation on the biosynthesis of polyphenols in tea seedslings. Plant Physiol. Biochem..

[24] Artem V., Antoce A.O., Namolosanu I., Ranca A., Petrescu A. (2015). The influence of the vine cultivation technology on the phenolic composition of red grapes. Horticulture.

[25] Zhu L., Zhang Y., Zhang W., Lu J. (2016). Effects of exogenous abscisic acid on phenolic characteristics of red *Vitis vinifera* grapes and wines. Food Sci. Biotechnol..

[26] Perestrelo R., Barros A.S., Rocha S.M., Câmara J.S. (2014). Establishment of the varietal profile of *Vitis vinifera* L. grape varieties from different geographical regions based on HS-SPME/GC-qMS combined with chemometric tools. Microchem. J..

[27] Silva J.K., Cazarin C.B.B., Correa L.C., Batista A.G., Furlan C.P.B., Biasoto A.C.T., Pereira G.E., Camargo A.C., Maróstica Junior M.R. (2016). Bioactive compounds of juices from two Brazilian grape cultivars. J. Sci. Food Agric..

[28] Heras-Roger J., Díaz-Romero C., Darias-Martín J. (2016). A comprehensive study of red wine properties according to variety. Food Chem..

[29] Wang S.Y., Camp M.J. (2000). Temperatures after bloom affect plant growth and fruit quality of strawberry. Sci. Hortic..

[30] Li X., Wasila H., Liu L., Yuan T., Gao Z., Zhao B., Ahmad I. (2015). Physicochemical characteristics, polyphenol compositions and antioxidant potential of pomegranate juices from 10 Chinese cultivars and the environmental factors analysis. Food Chem..

[31] Spayd S.E., Tarara J.M., Mee D.L., Ferguson J.C. (2012). Separation of sunlight and temperature effects on the composition of *Vitis vinifera* cv. Merlot berries. Am. J. Enol. Viticult..

[32] Sun X., Li L., Ma T., Liu X., Huang W., Zhan J. (2015). Profiles of phenolic acids and flavan-3-ols for select Chinese red wines: A comparison and differentiation according to geographic origin and grape variety. J. Food Sci..

[33] Yamane T., Shibayama K. (2006). Effects of trunk girdling and crop load levels on fruit quality and root elongation in ‘Aki Queen’ grapevines. J. Jap. Soc. Hortic. Sci..

[34] Roby G., Harbertson J.F., Adams D.A., Matthews M.A. (2004). Berry size and vine water deficits as factor in winegrape composition: Anthocyanins and tannins. Aust. J. Grape Wine R..

[35] Terry L.A., Chope G.A., Giné Bordonaba J. (2007). Effect of water deficit irrigation and inoculation with: Botrytis cinerea on strawberry (*Fragaria×ananassa*) fruit quality. J. Agric. Food Chem..

[36] Castellarin S.D., Matthews M.A., Gaspero G.D., Gambetta G.A. (2007). Water deficits accelerate ripening and induce changes in gene expression regulating flavonoid biosynthesis in grape berries. Planta.

[37] Doumett S., Fibbi D., Cincinelli A., Giordani E., Nin S., Del Bubba M. (2011). Comparison of nutritional and nutraceutical properties in cultivated fruits of *Fragaria vesca* L. produced in Italy. Food Res. Int..

[38] Guerrero-Chavez G., Scampicchio M., Andreotti C. (2015). Influence of the site altitude on strawberry phenolic composition and quality. Sci. Hortic..

[39] Wei Z., Zhao Y., Huang Y., Zhang Y., Lu J. (2015). Optimization of ultrasound-assisted extraction of ellagic acid and total phenols from muscadine (*Vitis rotundifolia*) by response surface methodology. Food Sci..

