# Supplementary material for: Profile of Polyphenol Compounds of Five Muscadine Grapes Cultivated in the United States and in Newly Adapted Locations in China
Source: Int J Mol Sci. 2017 Mar 14;18(3):631. doi: 10.3390/ijms18030631 (PMC5372644; doi:10.3390/ijms18030631)
Supplement: Supplementary file 1 [file ijms-18-00631-s001.zip › Table S4.pdf]

Table S4 Retention time (RT), Triple TOF-MS/MS m/z values, and formula of polyphenol compounds identified in five muscadine grape skins and seeds cultivated in different regions in the 2012 and 2013 seasons.[1]

Compounds	RT (min)	[M-H]- (m/z)	UPLC-Triple TOF-MS/MS (m/z) [2]	Formula
**Ellagic acids and precursors**				
Ellagic acid derivatives				
Ellagic acid-coumaroyl hexoside	6.40	609.1101	609.1186[M-H]-, 445.0438[M-H-Glc]-, 301.0001[M-H-Glc-Cou]-, 299.9914	C26H26O17
Ellagic acid glucoside	6.69	463.0520	463.0559[M-H]-, 301.0008[M-H-Glc]-, 299.9929	C20H16O13
Mono-*O*-methyl-ellagic acid rhamnoside	6.79	463.0537	415.1616, 299.0574, 298.0498, 284.0333, 283.0226, 269.1046, 256.0370, 161.0455	C22H22O11
Ducheside B	7.45	433.0408	433.0438, 300.9995, 299.9922	C19H14O12
Ducheside A	7.72	447.0567	447.0580[M-H]-, 300.9991, 299.9916	C20H16O12
Ellagic acid	8.28	300.9992	300.9986[M-H]-, 283.9956, 245.0083, 229.0137, 201.0186, 185.0238, 173.0236, 145.0290	C14H6O8
Ellagic acid-dihexoside	8.34	624.9959	300.9987	C23H14O21
Mono-*O*-methyl ellagic acid deoxyhexoside	8.12	461.0725	461.0352[M-H]-, 299.0168[M-H-Glc]-, 271.0265, 255.0280, 231.0293, 227.0370, 221.0459, 163.0026	C21H18O12
Ellagic acid derivative m/z 447	9.21	447.1823	447.1077[M-H]-, 301.0017, 299.9909, 298.9828, 285.0405, 284.0365, 227.0375, 175.0029, 151.0034	C27H28O6
Di-*O*-methyl ellagic acid-4-*O*-rhamnoside	9.30	475.0877	313.0395, 298.0145, 254.0239, 245.0426, 226.0258, 75.0097	C22H20O12
Mono-*O*-methyl-ellagic acid	10.08	315.0144	299.991[M-CH_3]-	C15H8O8
Galloyl-tri-*O*-methyl-ellagic acid hexoside	10.55	657.1127	657.1126[M-H]-, 491.0821, 343.0484, 329.0313, 328.0224, 314.0152, 313.0551, 287.0588, 169.0140	C30H26O17
Di-*O*-methyl ellagic acid	12.35	329.0306	314.0075, 298.9845, 270.9890, 242.9931	C16H10O8
Tri-*O*-methyl-ellagic acid	15.25	343.0462	328.0236, 313.0003, 297.9767, 285.0050, 269.9813	C17H12O8
Ellagitannins				
HHDP-glucose	1.62	481.0621	481.0637[M-H]-, 300.9988, 275.0201	C20H18O14
HHDP-galloyl-glucose (Isostrictinin)	3.22	633.0739	633.1528[M-H]-, 487.1357, 355.1228, 325.0773, 300.9988, 275.0209, 249.0403, 163.0249	C27H22O18
Galloyl-HHDP-hexose (Corilagin, Strictinin)	3.29	633.0738	633.0756[M-H]-, 487.1359, 309.1203, 300.9988[M-H-Gall-Hex]-, 275.0209, 169.0157[M-H-HHDP-Hex]-, 145.0256, 130.9983, 112.9882, 103.0038	C27H22O18
Ellagitannin m/z 633	3.35	633.1529	633.1545[M-H]-, 453.0648, 409.0765, 300.9982, 275.0242, 247.0243, 163.0251, 145.0139	C22H34O21
Pedunculagin α/ß isomer (Di-HHDP-glucose)	3.43	783.0684	783.0727[M-H]-, 481.0609[M-H-HHDP]-, 300.9990, 275.0210	C34H24O22
Eriodictyol coumaroyl hexose	3.61	595.1461	289.0715, 245.0810, 203.0837, 153.0191, 151.0030	C30H28O13
Ellagitannin m/z 689	3.97	689.1576	643.1553, 517.1244, 481.0998, 463.0902, 355.0680, 329.0889, 301.0360, 283.0251, 193.0142, 175.0039	C28H34O20
Ellagitannin m/z 643	4.05	643.1521	643.1555[M-H]-, 481.1009[M-H-Gall]-, 463.0935, 355.0683, 329.0890, 301.0360, 283.0260, 193.0151, 175.0044, 125.0250	C27H32O18
Ellagitannin m/z 681	4.12	681.1302	645.1669, 643.1601, 481.1047[M-H-Glc]-, 463.0884[M-H-H_2O]-, 355.0708, 301.0336[M-H-H_2O-Glc]-, 283.0303, 193.0145, 175.0030	C29H30O19
Ellagitannin m/z 671	4.14	671.1476	625.1435, 463.0892, 462.0808, 301.0344, 300.0262, 299.0190	C28H32O19
Ellagitannin m/z 497	4.39	497.0573	393.0498, 333.0295, 300.9961[M-H-H_2O-178]-, 298.9841, 273.0035, 247.0255, 245.0098, 203.0361	C20H18O15
Ellagitannin m/z 443	5.29	443.1922	443.1954[M-H]-, 237.1466, 189.1307, 119.0347, 101.0234, 89.0249, 71.0143	C21H32O10
Tellimagrandin I (Digalloyl-HHDP-glucose)	6.71	785.0848	785.0892[M-H]-, 300.9995, 275.0202	C34H26O22
Galloyl-bis-HHDP-glucose (Casuarictin)	6.97	935.0790	935.0831[M-H]-, 633.0771[M-H-HHDP]-, 300.9985[M-H-2HHDP]-	C41H28O26
Ellagitannin m/z 957	7.01	957.0911	957.0971[M-H]-, 897.0774, 821.0507, 783.0729, 763.0500, 701.0725, 481.0675, 301.0004	C33H34O33
Valoneic acid dilactone	7.02	469.0053	300.9997, 299.9898, 298.9864, 275.0032, 270.9903	C21H10O13
Ellagitannin m/z 831	7.54	831.0539	831.0599[M-H]-, 787.0685, 769.0581, 723.0518, 697.0780, 300.9988, 298.9847, 273.0024, 247.0248, 245.0094	C34H24O25
Isomallotinic acid	7.73	801.0784	783.0758, 765.0657, 649.0741[M-H-Gall]-, 615.0676, 613.0500, 597.0564, 300.9999, 298.9850	C34H26O23
Ellagitannin m/z 861	7.80	861.0655	815.0670, 797.0557, 779.0377, 751.0472, 733.0433, 435.0606, 301.0007, 298.9844, 291.0158, 247.0254	C35H26O26
Punicalin a/ß isomer	7.84	781.0528	781.0630[M-H]-, 763.0379, 753.0547, 745.0312, 735.0444, 451.0524, 449.0371, 300.9995, 298.9830	C34H22O22
Ellagitannin m/z 779	7.95	779.0432	751.0499[M-H-CO2]-, 733.0388, 449.0383[M-H-CO_2-HHDP]-, 300.9986, 298.9850, 286.9832[M-H-CO_2-HHDP-Hex-1]-, 275.0200, 261.0049, 214.9970, 145.0128	C27H24O27
Ellagitannin m/z 721	8.19	721.1061	419.1012[M-H-HHDP]-, 301.0003	C34H26O18
Tellimagrandin II (Trigalloyl-HHDP-glucose)	8.19	937.0972	937.1016[M-H]-, 785.0884[M-H-Gall]-, 635.0941, 300.9991	C41H30O26
Methyl brevifolin carboxylate	8.26	305.0305	305.0334[M-H]-, 273.0046, 245.0103, 229.0146, 201.0192, 189.0197, 173.0230, 145.0295, 133.0297, 117.0344	C14H10O8

Supplementary Table 4 *Cont.*

Compounds	RT (min)	[M-H]- (m/z)	UPLC-Triple TOF-MS/MS (m/z) [2]	Formula
Ellagitannin m/z 797	8.27	797.0475	751.0471, 733.0371, 300.9989, 298.9828, 275.0203, 273.0056, 247.0235, 217.0071	C34H22O23
Chebulagic acid	8.39	953.0895	953.1012[M-H]-, 935.0895[M-H-CO_2]-, 917.0796[M-H-2CO_2]-, 909.1158, 907.0906, 801.0811[M-H-Gall]-, 783.0761, 765.0640, 757.0926	C41H30O27
Ellagitannin m/z 603	8.80	603.0103	301.0001	C21H16O21
Ellagitannin m/z 967	9.08	967.1054	967.0950[M-H]-, 935.0805, 917.0677, 815.0909, 783.0718, 765.0623, 749.0672, 300.9987	C42H32O27
Gallagic acid	9.48	600.9909	600.9965[M-H]-, 314.9788, 300.9998, 298.9844, 270.9888	C28H10O16
Phloretin	13.19	273.0768	229.0856, 189.0553, 167.0334, 165.0222, 151.0036, 149.0288, 123.0445, 119.0499, 83.0138, 81.0343	C15H14O5
Precursors (Gallic acid derivatives)				
Tri-O-galloyl-glucose	1.15	635.0893	635.0893[M-H]-, 483.0790[M-H-Gall]-, 465.0685, 423.0578, 313.0562, 271.0450, 211.0242, 169.0141	C27H24O18
Mono-O-galloyl-glucose	2.37	331.0669	211.0283, 169.0140[M-H-Glc]-, 168.0054, 125.0244[M-H-Glc-CO_2]-, 124.0165	C13H16O10
Gallic acid	2.59	169.0145	125.0244[M-H-CO_2]-, 124.0168, 79.0196	C7H6O5
Di-*O*-galloyl quinic acid	4.58	495.0776	495.0815, 301.0007	C21H20O14
Di-O-galloyl-glucose	5.62	483.0781	483.0790[M-H]-, 447.0968, 331.0671[M-H-Gall]-, 313.0569, 271.0470, 211.0250, 169.0150[M-H-Gall-Glc]-, 125.0247	C20H20O14
Methyl gallate	5.86	183.0305	124.0165, 78.0118	C8H8O5
Gallotannin m/z 803	5.94	803.1885	803.1973[M-H]-, 641.1407[M-H-Gall]-, 495.1179[M-H-Gall-Cou]-, 477.1070[M-H-Gall-Hex-2]-, 355.0695, 333.0634, 315.0525, 297.0402, 288.9999, 193.0148	C33H40O23
Tetra-O-galloyl-glucose	7.75	787.0996	787.1025[M-H]-, 635.0923[M-H-Gall]-, 617.0817[M-H-Gall-H_2O]-, 465.0696 [M-H-2Gall-H2O]-, 169.0155635 [M-H-2Gall-H_2O-Gall-144]-	C34H28O22
Penta-O-galloyl-glucose	8.61	939.1110	939.1184[M-H]-, 787.1045[M-H-Gall]-, 769.0952[M-H-Gall-18]-, 635.0918 [M-H-Gall-18-134]-617.0824 [M-H-2Gall-18]-	C41H32O26
Gallic acid derivative m/z 569	9.07	569.2265	523.2199, 361.1665	C27H38O13
**Flavonols**				
Myricetin trimethylether	1.32	359.0761	359.0980[M-H]-, 344.0715, 181.0141, 143.0348, 139.0401, 119.0379, 85.0294	C18H16O8
Syringetin	1.81	345.0610	345.0839[M-H]-, 183.0303, 182.0236, 167.0351, 165.0177, 137.0240, 129.0194, 125.0241	C17H14O8
Amurensin	2.68	533.1667	487.1706, 293.0867, 179.0554, 163.0038, 103.0038, 89.0245, 59.0143	C26H30O12
Kaempferol acetyl glucoside	2.81	489.1043	489.1085[M-H]-, 323.0916	C23H22O12
Quercetin malonyl glucoside	3.94	549.0802	451.0560, 425.0747, 365.0541, 245.0079, 237.0401, 219.0303, 191.0356	C24H22O15
Quercetin-3,4'-diglucoside	4.13	625.1415	625.1457[M-H]-, 463.0907[M-H-Glc]-, 462.0827, 301.0354[M-H-2Glc]-, 300.0302, 299.0302	C27H30O17
Myricetin 3-*O*-glucoside	4.48	479.0837	479.0848[M-H]-, 299.0195[M-H-Glc]-, 190.9979, 163.0028	C21H20O13
Dihydroquercetin caffeoyl glucoside	4.70	627.1597	627.1625[M-H]-, 465.1063[M-H]-Glc]-, 447.0960, 339.0717, 355.0684, 285.0412, 267.0302, 193.0143	C27H32O17
Myricetin 3,4'-diglucoside	4.72	641.1371	641.1423[M-H]-, 479.0860[M-H-Glc]-, 317.0665[M-H-2Glc]-, 299.0201, 193.0144	C27H30O18
Kaempferol dihexose	4.73	609.1463	447.0957[M-H-Hex]-, 446.0875, 285.0405([M-H-2Hex]-, 284.0325, 283.0246	C27H30O16
Isorhamnetin sophorose	4.87	639.1573	639.1587[M-H]-, 477.1060, 476.0975, 315.0508, 314.0458, 313.0337, 300.0306	C28H32O17
Quercetin 3-*O*-glucoside	5.35	463.0886	463.0870[M-H]-, 300.0277[M-H-Glc]-, 301.0374, 299.9927	C21H20O12
Kaempferol 3-*O*-rhamnoside	5.97	431.0981	269.0438, 268.0391, 241.0530	C21H20O10
Kaempfeol-3-*O*-rutinoside	6.03	593.1515	355.1268, 321.0830, 193.0717, 169.0144, 161.0450, 143.0373	C27H30O15
Isorhamnetin 3-glucoside	6.22	477.1045	477.1059[M-H]-, 315.0525[M-H-Glc]-, 314.0440, 300.0275, 299.0190[M-H-Glc-CH_3]-, 283.0271[M-H-Glc-OH]-, 271.0268, 255.0285	C22H22O12
Laricitrin 3-*O*-glucoside	6.31	493.0988	493.1032[M-H]-, 447.0953, 285.0415, 331.0479[M-H-Glc]-, 313.0345, 284.0333, 205.0134, 189.9904	C22H22O13
Dihydroquercetin 3-*O*-hexoside	6.57	465.0620	465.0610[M-H]-, 303.0069[M-H-Glc]-, 301.9969[M-H-Glc]-, 300.9991, 299.9957	C27H14O8
Dihydrokaempferol 7-*O*-glucoside	6.59	449.1092	449.1130[M-H]-, 287.0619, 269.0514, 178.9992, 151.0047, 125.0259	C21H22O11
Myricetin 3-*O*-glucuronide	6.61	493.0639	493.1186[M-H]-, 447.0994, 437.0765, 331.0467, 298.0121, 285.0407, 125.0204	C21H18O14
Syringetin 3-glucoside	6.84	507.1157	507.1194[M-H]-, 461.1116, 345.0629[M-H-Glc]-, 331.0447, 299.0566, 297.0069, 190.9977	C23H24O13
Quercetin 3-glucuronide	7.01	477.0696	477.0679[M-H]-, 315.0177, 299.9913, 271.0240, 269.0449	C21H18O13
Rutin	8.09	609.1473	609.1510[M-H]-, 301.0368[M-H-Rha-Glc]-, 300.0290	C27H30O16
Dihydrokaempferol 3-*O*-arabinoside	8.15	419.0986	419.0995[M-H]-, 257.0455, 256.0383	C20H20O10
Laricitrin	9.16	331.0470	300.0302, 271.0266, 255.0303, 243.0306, 215.0347, 178.9978, 151.0028, 107.0140	C16H12O8
Isorhamnetin 3-glucuronide	9.39	491.0851	491.0908[M-H]-, 313.0005, 285.0112	C22H20O13
Kaempferol 3-*O*-glucuronide	9.65	461.0726	461.0600[M-H]-, 315.0143, 299.9916, 255.0280, 227.0340, 221.0469	C21H18O12
Kaempferol 3-*O*-glucoside	9.66	447.0945	447.0942[M-H]-, 285.0409[M-H-Glc]-, 284.0334, 257.0452, 151.0041	C21H20O11

Supplementary Table 4 *Cont.*

Compounds	RT (min)	[M-H]- (m/z)	UPLC-Triple TOF-MS/MS (m/z) [2]	Formula
Myricetin	10.15	317.0306	317.0308[M-H]-, 271.0257, 178.9980, 151.0036, 137.0242, 109.0293, 107.0134	C15H10O8
Dihydroquercetin	10.75	303.0512	153.0180, 151.0395, 123.0453, 105.0350	C15H12O7
Quercetin	12.10	301.0356	301.0361[M-H]-, 178.9983, 151.0034, 121.0286, 107.0137	C15H10O7
Kaempferol	13.81	285.0405	285.0401[M-H]-, 229.0502, 211.0391, 187.0393, 185.0602	C15H10O6
Isorhamnetin	13.96	315.0512	315.0528[M-H]-, 300.0276, 283.0287, 271.0253, 255.0292, 151.0024, 107.0148	C16H12O7
**Benzoic acids**				
Malic acid	1.37	133.0138	78.9625, 71.0143	C4H6O5
Citric acid	1.67	191.0199	111.0086, 87.0090, 85.0304, 59.0134, 57.0355	C6H8O7
Mucic acid methyl ester gallate	2.32	375.0570	205.0380, 169.0170, 111.0097, 87.0088	C14H16O12
Mucic acid methyl ester digallate	2.34	527.0675	451.0552, 423.0627, 391.0349, 303.0155, 288.9978, 275.0191, 249.0421, 245.0083	C21H20O16
Mucic acid digallate	3.19	513.0520	433.0460, 363.0352, 261.0010, 245.0101, 209.0079[M-H-2Gall]-, 191.0355[M-H-2Gall-H_2O]-, 169.0151	C20H18O16
Vanillic acid	3.23	167.0352	137.0201, 136.0173, 122.0384, 109.0305, 95.0514, 91.0175	C8H8O4
Mucic acid lactone digallate	3.24	495.0415	423.0589, 379.0708, 363.0349, 261.0052, 245.0455, 243.0322, 235.0260, 191.0353	C20H16O15
Vanillic acid 4-glucoside	3.25	329.0878	329.0882[M-H]-, 167.0349, 166.0272, 151.0402, 109.0293	C14H18O9
1-*O*-protocatechuoyl-glucose	3.92	315.0737	207.0001, 168.9397, 153.0192[M-H-Hex]-, 152.0074, 109.0295, 108.0230	C13H16O9
Brevifolin carboxylic acid	6.73	291.0150	247.0257, 219.0301, 191.0350, 173.0238, 1450.292	C13H8O8
Dihydroxybenzoic acid (Genestic acid)	7.19	153.0193	109.0288, 108.0211, 91.0180, 81.0340, 65.0049	C7H6O4
Syringic acid	7.96	197.0458	182.0227, 169.0158, 166.9973, 162.8394, 160.8408, 125.0236, 124.0159, 123.0080	C9H10O5
Dihydroxybenzate ethyl ester	10.54	181.0509	166.0278, 149.0243	C9H10O4
Mono hydroxybenzoic acid (Salicylic acid)	11.37	137.0244	136.8610, 93.0327, 75.0248, 65.0428	C7H6O3
**Flavan-3-ols**				
(Epi)gallocatechin	3.72	305.0664	153.0187[M-H-Gall]-, 151.0040, 125.0246, 121.0284, 107.0139	C15H14O7
(Epi)catechin hexoside	5.05	451.1243	361.0920, 331.0827, 313.0718, 289.0719[M-H-Hex]-, 287.0932, 269.0809, 245.0818, 205.0512, 203.0709	C21H24O11
(Epi)gallocatechin gallate	5.73	457.0781	331.0846, 303.0505[M-2H-Gall]-, 169.0133, 153.0190, 150.9999, 151.0399 [M-H-Epigallocatechin]-, 125.0230, 123.0091	C22H18O11
Procyanidin B	5.93	577.1349	425.0894 [M-H-Gall]-, 407.0787, 289.0724[M-H-Epicat]-, 273.0412, 245.0817, 125.0251	C30H26O12
(Epi)catechin	6.59	289.0716	245.0825[M-H-CO_2]-, 203.0714, 151.0401, 137.0249, 125.0245, 123.0461, 109.0306	C15H14O6
Procyanidin trimer	6.77	865.1992	865.2036[M-H]-, 713.1533[M-H-Gall]-, 695.1404[M-H-Gall-18]-, 577.1379[M-H-Epicat]-, 575.1216, 425.0871, 407.0773, 289.0718[M-H-2Epicat]-, 285.0557	C45H38O18
Propelargonidin dimer	7.43	561.1404	561.1429[M-H]-, 435.1087, 423.1092, 405.0977, 273.0398, 271.0607, 229.0511, 173.0601, 137.0234, 125.0240	C30H26O11
Propelargonidin trimer	7.53	849.2035	849.2079[M-H]-, 679.1609, 653.1706, 561.1417, 545.1124, 431.0787, 407.0778, 271.0592	C45H38O17
(Epi)catechin gallate	8.29	441.0825	289.0722[M-H-Gall]-, 245.0819[M-H-Gall-CO_2]-, 203.0715, 169.0148, 125.0248	C22H18O10
Methylepigallocatechin-3-gallate	8.65	471.0933	287.0570, 269.0463, 169.0147, 165.0190, 161.0246, 151.0398, 125.0253	C23H20O11
Procyanidin A	9.61	575.1189	407.0769, 289.0731, 287.0525[M-H-Epicat]-, 285.0424, 169.0155, 165.0222, 137.0243, 125.0239	C30H24O12
**Stilbens**				
Resveratrol	10.90	227.0716	227.0706, 185.0599, 183.0793, 182.0728, 181.0654, 143.0499, 117.0343, 115.0549	C14H12O3
Resveratrol-3-*O*-glucoside	12.33	389.1247	329.0998, 269.0788, 240.0754, 223.0812, 212.0825, 180.0551, 147.0560	C20H22O8
**Cinnamic acids**				
Di-coumaroyl hexose	2.25	471.1307	355.1247, 247.0226, 193.0675, 163.0250, 145.0144, 112.9886, 103.0041	C24H24O10
Ferulic acid	10.60	193.0511	193.0547, 161.0246, 134.0379, 133.0288, 59.0157	C10H10O4

[1] Peak View 2.0 and Marker View 1.2.1 software (AB SCIEX, USA) are used for qualitative analysis. The polyphenol database of both offline self-built and online Chemspider (http://www.chemspider.com), Mass Bank (http://www.massbank.jp) and Phenol-explorer (http://phenol-explorer.eu) are used for searching and comparing, together with the RT and MS/MS ions information described in references.

[2] The MS/MS response ions are shown with signal strength ≥ 10%. Only the top ten ions are exhibited if the ion number ≥ 10. The square brackets represent fragmentation pattern of the deprotonated ions. Glc: glucose; Cou: coumaroyl; Gall: galloyl; Hex: hexose; HHDP: hexahydroxydiphenic acid; Rha: rhamnoside; Epicat: epicatechin.
