# Supplementary material for: Profile of Polyphenol Compounds of Five Muscadine Grapes Cultivated in the United States and in Newly Adapted Locations in China
Source: Int J Mol Sci. 2017 Mar 14;18(3):631. doi: 10.3390/ijms18030631 (PMC5372644; doi:10.3390/ijms18030631)
Supplement: Supplementary file 1 [file ijms-18-00631-s001.zip › ijms-178535-suppl-figures.pdf]

Profile of Polyphenol Compounds of Five Muscadine Grapes Cultivated in the United States and in Newly Adapted Locations in China

Zheng Wei, Jianming Luo, Yu Huang, Wenfeng Guo, Yali Zhang, Huan Guan, Changmou Xu and Jiang Lu

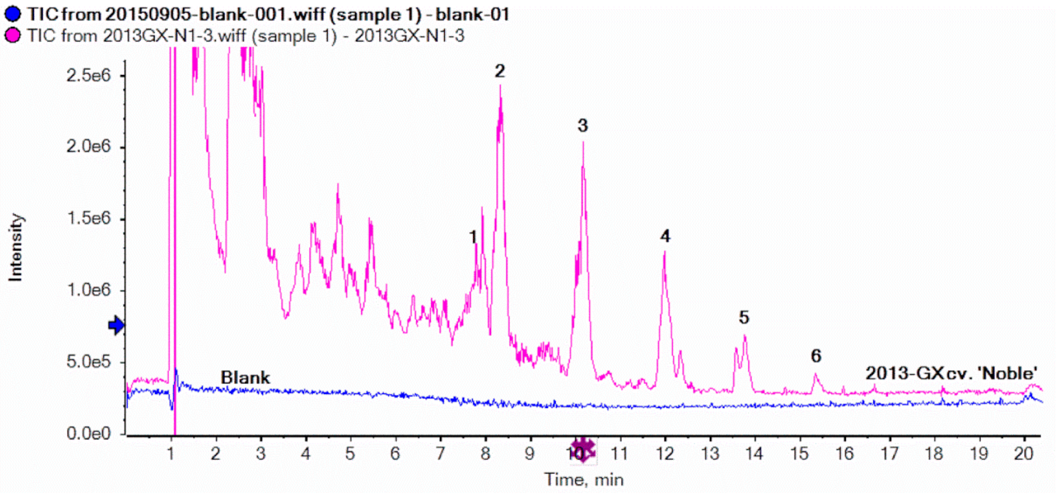

Figure S1. The total ion chromatogram (TIC) of blank and skin Guangxi cv. 'Noble' in 2013 season. Polyphenols: 1 = Ducheside A; 2 = Ellagic acid; 3 = Myricetin; 4 = Quercetin; 5 = Kaempferol; 6 = Tri-*O*-methyl-ellagic acid.

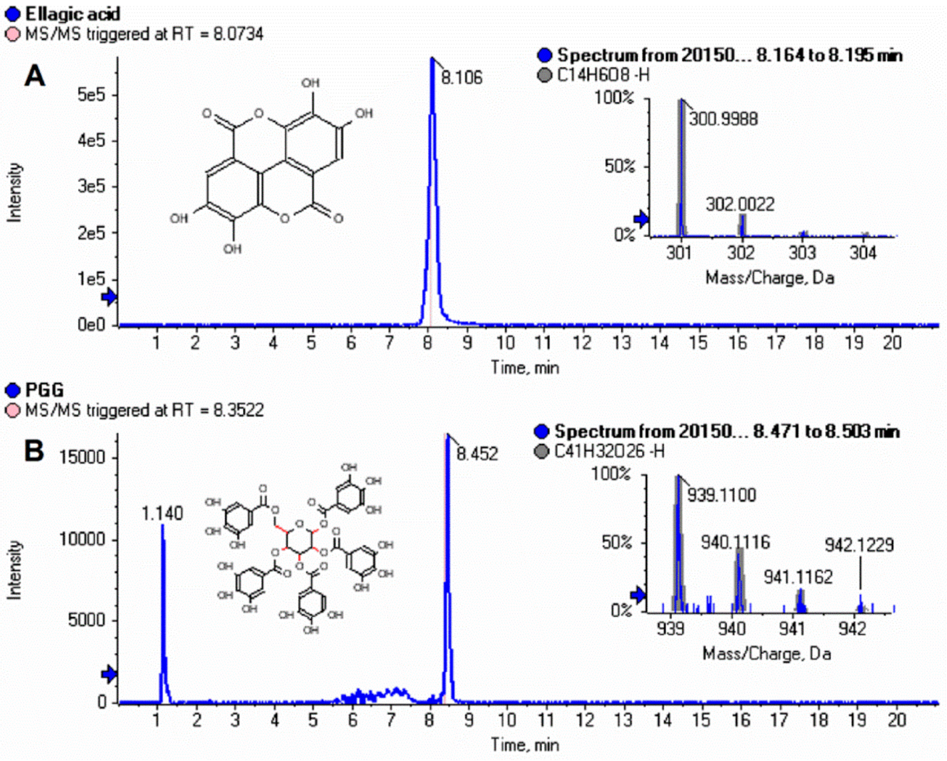

Figure S2. The retention time, molecular structural formula and isotope deprotonated ions of Ellagic acid (A) and Penta-*O*-galloyl-glucose (PGG) (B) obtained by ultra performance liquid chromatography tandem triple quadrupole time-of-flight mass spectrometry (UPLC Triple TOF-MS/MS).

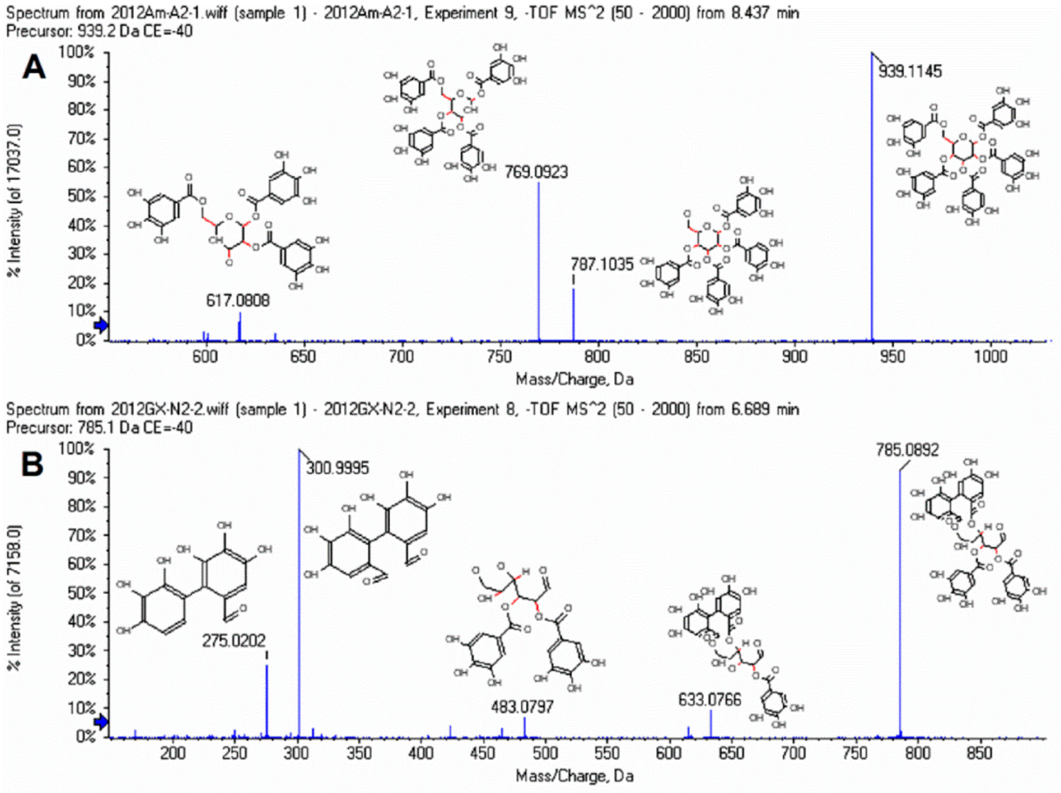

Figure S3. The fragmentation pattern of deprotonated ions of Penta-*O*-galloyl-glucose (PGG) (A) and Tellimagrandin I (B) obtained by UPLC Triple TOF-MS/MS.
