# Supplementary material for: Profile of Polyphenol Compounds of Five Muscadine Grapes Cultivated in the United States and in Newly Adapted Locations in China
Source: Int J Mol Sci. 2017 Mar 14;18(3):631. doi: 10.3390/ijms18030631 (PMC5372644; doi:10.3390/ijms18030631)
Supplement: Supplementary file 1 [file ijms-18-00631-s001.zip › Table S1.pdf]

Table S1. Total and individual polyphenol concentrations identified in skins and seeds of five muscadine grapes from Tallahassee-Florida in the 2012 season.[1]

**Compounds** [2]	**Noble**		**Alachua**		**Carlos**		**Fry**		**Granny Val**	
	**skins**	**seeds**	**skins**	**seeds**	**skins**	**seeds**	**skins**	**seeds**	**skins**	**seeds**
**Ellagic acids and precursors (mg GAE/100g DW)**										
Ellagic acid derivatives										
Ellagic acid	112.11 ± 11.56	175.98 ± 4.64	96.93 ± 24.74	52.72 ± 4.91	91.63 ± 4.84	26.94 ± 2.64	76.18 ± 27.59	30.51 ± 4.16	51.71 ± 5.33	10.33 ± 0.75
Mono-*O*-methyl-ellagic acid	3.11 ± 2.06	14.82 ± 1.33	2.86 ± 0.23	7.92 ± 1.08	2.54 ± 0.30	5.47 ± 0.35	5.23 ± 1.76	4.59 ± 0.52	3.07 ± 1.66	1.26 ± 0.10
Mono-*O*-methyl-ellagic acid rhamnoside	0.36 ± 0.16	0.53 ± 0.00	0.34 ± 0.21	0.53 ± 0.00	nd	0.53 ± 0.00	nd	nd	nd	nd
Mono-*O*-methyl-ellagic acid deoxyhexoside	0.23 ± 0.09	nd	0.16 ± 0.05	nd	0.11 ± 0.00	nd	0.11 ± 0.00	nd	nd	nd
Ducheside A	2.41 ± 0.95	16.46 ± 2.88	3.67 ± 2.65	6.39 ± 0.63	8.88 ± 1.70	4.59 ± 0.44	5.41 ± 5.15	4.14 ± 0.65	2.99 ± 2.87	1.63 ± 0.06
Ducheside B	5.35 ± 2.55	5.27 ± 0.93	12.31 ± 1.66	0.74 ± 0.04	6.57 ± 5.61	0.84 ± 0.03	5.74 ± 4.59	nd	4.86 ± 3.93	0.92 ± 0.04
Di-*O*-methyl-ellagic acid	0.64 ± 0.21	8.05 ± 0.85	0.61 ± 0.23	2.57 ± 0.08	0.35 ± 0.02	1.28 ± 0.03	1.36 ± 0.15	1.39 ± 0.04	0.86 ± 0.18	0.88 ± 0.02
Di-*O*-methyl-ellagic acid 4-*O*-rhamnoside	0.18 ± 0.05	nd	0.15 ± 0.04	nd	nd	nd	nd	nd	nd	nd
Tri-*O*-methyl-ellagic acid	8.19 ± 0.16	7.42 ± 2.47	6.99 ± 2.40	5.77 ± 1.16	3.75 ± 0.09	6.65 ± 0.80	13.76 ± 0.65	5.59 ± 1.19	7.01 ± 1.50	3.48 ± 0.07
Galloyl-tri-*O*-methyl-ellagic acid hexoside	nd	0.72 ± 0.03	0.11 ± 0.00	0.76 ± 0.02	nd	nd	nd	nd	nd	nd
Ellagic acid glucoside	3.97 ± 1.55	3.95 ± 0.04	5.53 ± 2.18	2.66 ± 0.03	4.05 ± 0.86	1.79 ± 0.12	3.00 ± 0.24	2.21 ± 0.22	2.14 ± 0.38	0.79 ± 0.04
Ellagic acid-dihexoside	nd	0.76 ± 0.03	0.26 ± 0.04	nd	0.20 ± 0.04	nd	0.23 ± 0.04	nd	0.24 ± 0.04	nd
Ellagic acid derivative m/z 447	0.36 ± 0.17	nd	nd	nd	nd	nd	nd	nd	0.18 ± 0.02	nd
Ellagitannins										
Ellagitannin m/z 443	0.19 ± 0.04	0.83 ± 0.06	0.24 ± 0.06	0.86 ± 0.07	0.65 ± 0.11	0.85 ± 0.04	0.39 ± 0.05	0.76 ± 0.04	0.44 ± 0.14	0.69 ± 0.02
Phloretin	nd	2.00 ± 0.19	nd	3.01 ± 0.21	nd	2.77 ± 0.10	0.11 ± 0.00	3.39 ± 0.30	nd	1.72 ± 0.14
Methyl brevifolin carboxylate	5.80 ± 0.17	13.03 ± 0.81	7.46 ± 2.52	9.39 ± 3.23	5.43 ± 1.37	2.68 ± 0.05	7.53 ± 0.43	5.00 ± 0.34	3.93 ± 2.07	0.93 ± 0.05
Valoneic acid dilactone	0.46 ± 0.04	nd	0.44 ± 0.18	nd	0.33 ± 0.02	nd	0.50 ± 0.15	nd	0.52 ± 0.17	nd
HHDP glucose	0.76 ± 0.22	3.61 ± 0.21	1.27 ± 0.65	0.92 ± 0.02	1.56 ± 0.15	0.65 ± 0.01	0.98 ± 0.68	0.96 ± 0.06	0.40 ± 0.06	nd
Ellagitannin m/z 497	0.19 ± 0.06	nd	0.21 ± 0.18	nd	1.46 ± 0.06	nd	0.79 ± 0.57	nd	0.92 ± 0.72	nd
Eriodictyol coumaroyl hexose	nd	nd	nd	0.77 ± 0.14	nd	nd	nd	nd	nd	nd
Gallagic acid	0.42 ± 0.07	nd	0.42 ± 0.06	nd	0.36 ± 0.02	nd	0.41 ± 0.12	nd	0.36 ± 0.09	nd
Ellagitannin m/z 603	4.14 ± 0.35	nd	3.74 ± 0.84	0.40 ± 0.24	3.06 ± 0.12	0.43 ± 0.17	2.88 ± 0.90	0.46 ± 0.12	nd	nd
Galloyl-HHDP-glucose (Corilagin, Strictinin)	0.20 ± 0.01	2.70 ± 1.01	0.31 ± 0.08	1.14 ± 0.45	0.15 ± 0.13	nd	nd	0.95 ± 0.09	0.14 ± 0.03	nd
HHDP-galloyl-glucose (Isostrictinin)	0.30 ± 0.17	1.93 ± 0.44	0.25 ± 0.10	0.88 ± 0.01	0.25 ± 0.07	nd	nd	0.80 ± 0.16	0.17 ± 0.05	nd
Ellagitannin m/z 633	0.34 ± 0.29	nd	0.23 ± 0.20	nd	0.20 ± 0.06	nd	nd	nd	0.15 ± 0.03	nd
Ellagitannin m/z 643	11.88 ± 3.62	nd	13.94 ± 3.65	nd	0.26 ± 0.14	nd	nd	nd	nd	nd
Ellagitannin m/z 671	1.59 ± 0.12	nd	2.01 ± 0.41	nd	0.12 ± 0.00	nd	nd	nd	nd	nd
Ellagitannin m/z 681	1.79 ± 0.52	nd	2.54 ± 1.18	nd	0.13 ± 0.02	nd	nd	nd	nd	nd
Ellagitannin m/z 689	1.26 ± 0.34	nd	1.68 ± 0.42	nd	nd	nd	nd	nd	nd	nd
Ellagitannin m/z 721	0.38 ± 0.12	nd	0.38 ± 0.19	nd	nd	nd	nd	nd	nd	nd

Supplementary Table 1 ***Cont.***

Compounds [2]	Noble		Alachua		Carlos		Fry		Granny Val	
	**skins**	**seeds**	**skins**	**seeds**	**skins**	**seeds**	**skins**	**seeds**	**skins**	**seeds**
Ellagitannin m/z 779	0.45 ± 0.15	nd	nd	nd	0.76 ± 0.12	nd	0.36 ± 0.06	nd	nd	nd
Punicalin α/ß isomer	nd	nd	nd	nd	0.23 ± 0.09	nd	nd	nd	nd	nd
Pedunculagin α/ß isomer (Di-HHDP-glucose)	nd	0.74 ± 0.01	nd	nd	nd	nd	nd	nd	nd	nd
Tellimagrandin I (Digalloyl-HHDP-glucose)	nd	4.84 ± 0.30	nd	1.93 ± 0.36	nd	1.08 ± 0.03	nd	1.91 ± 0.40	nd	0.69 ± 0.02
Ellagitannin m/z 797	0.23 ± 0.02	nd	0.26 ± 0.07	nd	0.39 ± 0.11	nd	nd	nd	0.36 ± 0.15	nd
Isomallotinic acid	nd	0.79 ± 0.02	nd	nd	nd	nd	nd	nd	nd	nd
Ellagitannin m/z 831	0.34 ± 0.12	nd	nd	nd	1.33 ± 0.34	nd	nd	nd	nd	nd
Ellagitannin m/z 861	0.27 ± 0.04	nd	nd	nd	0.47 ± 0.15	nd	nd	nd	0.28 ± 0.05	nd
Galloyl-bis-HHDP-glucose (Casuarinin)	nd	0.96 ± 0.02	nd	nd	nd	nd	nd	nd	nd	nd
Tellimagrandin II (Trigalloyl-HHDP-glucose)	nd	0.99 ± 0.04	nd	0.97 ± 0.07	nd	0.57 ± 0.01	nd	0.95 ± 0.07	nd	0.54 ± 0.00
Ellagitannin m/z 957	0.23 ± 0.07	nd	0.37 ± 0.17	nd	0.32 ± 0.03	nd	0.57 ± 0.18	nd	0.22 ± 0.06	nd
Ellagitannin m/z 967	nd	0.80 ± 0.06	nd	nd	nd	0.65 ± 0.07	nd	nd	nd	nd
Precursors (Gallic acid derivatives)										
Gallic acid	2.71 ± 0.50	68.67 ± 5.51	4.35 ± 1.68	69.83 ± 7.22	5.37 ± 0.09	43.84 ± 4.90	3.49 ± 0.99	47.29 ± 3.10	2.80 ± 0.98	20.05 ± 2.94
Methyl gallate	2.13 ± 0.67	41.16 ± 2.39	2.48 ± 1.07	36.05 ± 13.28	1.70 ± 0.32	51.12 ± 12.80	2.02 ± 1.15	53.13 ± 11.14	1.31 ± 0.88	31.78 ± 1.39
Di-*O*-galloyl quinic acid	0.10 ± 0.01	nd	0.17 ± 0.08	nd	0.23 ± 0.13	nd	0.17 ± 0.03	nd	0.08 ± 0.01	nd
Mono-*O*-galloyl-glucose	0.48 ± 0.11	2.80 ± 0.46	0.64 ± 0.08	3.59 ± 1.21	0.88 ± 0.44	2.12 ± 0.73	0.65 ± 0.03	2.94 ± 0.28	nd	1.02 ± 0.43
Di-*O*-galloyl-glucose	0.27 ± 0.06	0.67 ± 0.03	0.57 ± 0.17	0.67 ± 0.02	0.83 ± 0.05	0.59 ± 0.04	0.43 ± 0.12	0.66 ± 0.14	0.30 ± 0.09	0.58 ± 0.01
Tri-*O*-galloyl-glucose	nd	3.68 ± 0.27	nd	3.06 ± 0.95	0.11 ± 0.01	1.74 ± 0.55	nd	1.81 ± 0.46	nd	1.06 ± 0.10
Tetra-*O*-galloyl-glucose	nd	13.93 ± 2.40	nd	14.98 ± 0.23	nd	7.83 ± 0.42	nd	15.67 ± 0.84	nd	5.30 ± 0.56
Penta-*O*-galloyl-glucose	nd	238.65 ±16.05	nd	282.25 ± 56.06	nd	92.18 ± 7.05	nd	297.39 ± 21.66	nd	69.52 ± 18.35
Gallic acid derivative m/z 569	nd	nd	nd	nd	0.27 ± 0.06	nd	nd	nd	nd	nd
Gallotannin m/z 803	0.48 ± 0.13	nd	0.42 ± 0.29	nd	nd	nd	nd	nd	nd	nd
Total	174.3 ± 19.48 [d]	636.75 ± 28.83 [a]	174.31 ± 26.69 [d]	510.76 ± 39.46 [b]	144.94 ± 5.20 [d]	257.21 ± 20.16 [c]	132.3 ± 40.12 [d]	482.5 ± 8.70 [b]	85.42 ± 7.44 [e]	153.14 ± 22.68 [d]
**Flavonols (mg RE/100g DW)**										
Myricetin	nd	0.02 ± 0.02	nd	nd	0.04 ± 0.02	nd	nd	nd	nd	nd
Myricetin tri-methyl ether	12.68 ± 3.99	2.11 ± 0.84	12.27 ± 2.02	nd	15.29 ± 3.19	nd	18.80 ± 0.50	nd	13.84 ± 7.35	nd
Myricetin 3-*O*-glucuronide	nd	0.61 ± 0.03	nd	nd	nd	nd	nd	nd	nd	nd
Myricetin 3-*O*-glucoside	1.10 ± 0.45	nd	1.29 ± 0.08	nd	0.16 ± 0.02	nd	0.12 ± 0.02	nd	nd	nd
Myricetin 3,4'-diglucoside	0.28 ± 0.04	nd	0.37 ± 0.11	nd	nd	nd	nd	nd	nd	nd
Quercetin	1.11 ± 0.44	0.46 ± 0.24	2.47 ± 2.42	0.06 ± 0.05	2.35 ± 0.49	nd	0.44 ± 0.24	0.10 ± 0.06	nd	nd
Quercetin 3-glucuronide	0.35 ± 0.10	nd	0.23 ± 0.06	nd	0.23 ± 0.10	nd	0.14 ± 0.01	nd	0.14 ± 0.02	nd
Quercetin 3-*O*-glucoside	1.33 ± 0.04	0.17 ± 0.01	1.28 ± 0.63	0.13 ± 0.02	0.28 ± 0.04	0.12 ± 0.01	nd	0.12 ± 0.03	nd	0.09 ± 0.00
Quercetin malonyl glucoside	0.07 ± 0.00	nd	nd	nd	0.08 ± 0.01	nd	nd	nd	nd	nd

Supplementary Table 1 ***Cont.***

Compounds [2]	Noble		Alachua		Carlos		Fry		Granny Val	
	skins	seeds	skins	seeds	skins	seeds	skins	seeds	skins	seeds
Rutin	0.08 ± 0.01	nd	0.08 ± 0.00	nd	nd	nd	nd	nd	nd	nd
Quercetin 3,4'-diglucoside	1.56 ± 0.21	nd	2.01 ± 0.35	nd	nd	nd	nd	nd	nd	nd
Dihydroquercetin	nd	8.19 ± 1.59	nd	14.55 ± 5.91	nd	15.02 ± 1.08	nd	21.69 ± 3.19	nd	6.84 ± 1.54
Dihydroquercetin 3-*O*-hexoside	nd	nd	nd	nd	nd	nd	0.24 ± 0.02	nd	nd	nd
Dihydroquercetin caffeoyl glucoside	5.15 ± 2.31	nd	4.45 ± 3.31	nd	0.09 ± 0.00	nd	nd	nd	nd	nd
Kaempferol	0.18 ± 0.22	nd	0.26 ± 0.25	nd	0.10 ± 0.05	nd	nd	nd	nd	nd
Kaempferol 3-*O*-glucuronide	0.11 ± 0.03	nd	0.22 ± 0.18	nd	0.19 ± 0.02	nd	0.18 ± 0.07	nd	nd	nd
Kaempferol 3-*O*-rhamnoside	0.69 ± 0.17	nd	1.23 ± 0.37	nd	1.78 ± 0.09	nd	2.57 ± 1.16	nd	1.82 ± 1.09	nd
Kaempferol 3-*O*-glucoside	0.69 ± 0.51	1.32 ± 0.74	0.59 ± 0.48	nd	nd	nd	nd	nd	nd	nd
Amurensin	nd	nd	nd	nd	0.08 ± 0.00	nd	nd	nd	nd	nd
Kaempferol dihexose	0.68 ± 0.24	nd	0.69 ± 0.34	nd	nd	nd	nd	nd	nd	nd
Kaempferol 3-*O*-rutinoside	0.10 ± 0.02	nd	0.11 ± 0.04	nd	nd	nd	0.17 ± 0.06	nd	nd	nd
Dihydrokaempferol 3-O-arabinoside	1.75 ± 0.72	nd	1.59 ± 0.87	nd	0.09 ± 0.00	nd	0.09 ± 0.00	nd	nd	nd
Dihydrokaempferol 7-*O*-glucoside	nd	1.34 ± 0.05	nd	1.53 ± 0.22	nd	1.33 ± 0.14	nd	1.11 ± 0.05	nd	0.94 ± 0.11
Isorhamnetin	nd	4.48 ± 0.75	0.44 ± 0.43	3.74 ± 0.37	0.28 ± 0.03	3.45 ± 0.09	nd	4.78 ± 0.34	nd	2.31 ± 0.12
Isorhamnetin 3-glucuronide	0.08 ± 0.00	0.18 ± 0.05	nd	nd	0.20 ± 0.02	nd	0.07 ± 0.00	nd	0.09 ± 0.02	nd
Isorhamnetin 3-glucoside	0.94 ± 0.23	nd	1.12 ± 0.72	nd	nd	nd	nd	nd	nd	nd
Isorhamnetin sophorose	0.76 ± 0.24	nd	0.63 ± 0.11	nd	0.08 ± 0.01	nd	nd	nd	nd	nd
Laricitrin	nd	0.09 ± 0.00	nd	nd	nd	0.09 ± 0.01	0.12 ± 0.06	0.08 ± 0.01	0.09 ± 0.02	nd
Laricitrin 3-*O*-glucoside	1.22 ± 0.28	nd	1.18 ± 0.57	nd	0.10 ± 0.00	nd	0.08 ± 0.00	nd	0.10 ± 0.02	nd
Syringetin	4.49 ± 3.20	nd	5.22 ± 2.21	1.25 ± 0.93	nd	0.68 ± 0.31	7.03 ± 1.06	nd	nd	nd
Total	35.42 ± 3.12 [a,b]	18.96 ± 2.65 [e]	37.74 ± 4.01 [a]	21.26 ± 5.79 [d,e]	21.52 ± 3.61 [d,e]	20.69 ± 0.85 [d,e]	30.06 ± 2.11 [b,c]	27.89 ± 3.47 [c,d]	16.07 ± 8.41 [e,f]	10.17 ± 1.77 [f]
**Benzoic acids (mg GAE/100g DW)**										
Vanillic acid	0.46 ± 0.24	nd	0.37 ± 0.04	nd	nd	0.55 ± 0.02	nd	0.54 ± 0.00	nd	0.54 ± 0.00
Vanillic acid 4-glucoside	5.67 ± 1.96	nd	2.20 ± 1.26	nd	nd	nd	0.16 ± 0.02	0.54 ± 0.00	0.14 ± 0.02	0.54 ± 0.01
Syringic acid	0.12 ± 0.01	1.67 ± 0.10	0.15 ± 0.02	4.12 ± 0.03	nd	2.71 ± 0.12	nd	5.41 ± 0.29	nd	0.60 ± 0.00
Mono-hydroxybenzoic acid	0.20 ± 0.04	0.53 ± 0.00	0.21 ± 0.03	nd	0.26 ± 0.02	0.53 ± 0.00	0.20 ± 0.02	0.53 ± 0.00	0.20 ± 0.02	0.53 ± 0.00
Di-hydroxybenzoic acid	0.46 ± 0.11	0.59 ± 0.01	0.37 ± 0.22	0.55 ± 0.01	0.60 ± 0.06	0.57 ± 0.01	0.56 ± 0.13	0.54 ± 0.01	0.79 ± 0.20	0.55 ± 0.00
Brevifolin carboxylic acid	10.76 ± 4.02	0.66 ± 0.07	11.54 ± 2.83	0.66 ± 0.05	7.92 ± 0.67	0.57 ± 0.01	10.00 ± 2.03	0.60 ± 0.01	9.98 ± 1.49	0.57 ± 0.02
Mucic acid digallate	0.67 ± 0.20	nd	0.53 ± 0.13	nd	1.20 ± 0.31	nd	0.57 ± 0.05	nd	0.48 ± 0.03	nd
Mucic acid lactone digallate	0.60 ± 0.10	nd	0.63 ± 0.20	nd	0.99 ± 0.17	nd	0.55 ± 0.07	nd	0.52 ± 0.14	nd
Mucic acid methyl ester digallate	0.57 ± 0.14	nd	0.79 ± 0.32	nd	0.75 ± 0.19	nd	0.74 ± 0.12	nd	0.75 ± 0.22	nd
Citric acid	nd	0.75 ± 0.09	0.25 ± 0.13	1.42 ± 0.98	0.33 ± 0.05	nd	0.37 ± 0.10	nd	3.28 ± 5.30	0.63 ± 0.02
Malic acid	nd	nd	nd	nd	0.24 ± 0.11	nd	0.16 ± 0.04	nd	0.28 ± 0.13	nd
1-*O*-protocatechuoyl-glucose	nd	nd	nd	nd	nd	nd	nd	0.98 ± 0.08	nd	0.98 ± 0.03
Total	19.49 ± 2.43 [a]	4.20 ± 0.25 [f]	17.04 ± 2.40 [b]	6.75 ± 1.01 [e,f]	12.28 ± 0.91 [c,d]	4.93 ± 0.14 [e,f]	13.31 ± 2.38 [b,c,d]	9.14 ± 0.37 [d,e]	16.41 ± 6.13 [a,b,c]	4.93 ± 0.03 [e,f]

Supplementary Table 1 ***Cont.***

Compounds [2]	Noble		Alachua		Carlos		Fry		Granny Val	
	**skins**	**seeds**	**skins**	**seeds**	**skins**	**seeds**	**skins**	**seeds**	**skins**	**seeds**
**Flavan-3-ols (mg EE/100g DW)**										
(Epi)catechin	nd	47.39 ± 10.27	0.02 ± 0.00	120.14 ± 25.58	0.02 ± 0.00	116.49 ± 6.81	0.02 ± 0.00	149.5 ± 32.53	0.02 ± 0.00	135.26 ± 15.14
(Epi)catechin gallate	nd	12.70 ± 2.50	nd	32.00 ± 3.59	nd	27.53 ± 3.93	nd	44.8 ± 1.16	nd	35.53 ± 1.13
(Epi)catechin hexoside	nd	3.28 ± 0.53	nd	5.20 ± 3.92	nd	4.01 ± 2.33	nd	5.49 ± 1.86	nd	1.90 ± 0.67
(Epi)gallocatechin	nd	nd	nd	1.18 ± 0.40	nd	nd	nd	0.96 ± 0.15	nd	1.02 ± 0.07
(Epi)gallocatechin gallate	nd	2.32 ± 0.16	nd	2.48 ± 0.87	nd	2.24 ± 0.73	nd	2.08 ± 0.55	nd	1.67 ± 0.21
Methyl epigallocatechin -3-gallate	nd	21.46 ± 3.47	nd	21.52 ± 4.54	0.03 ± 0.01	25.04 ± 3.36	nd	22.25 ± 2.44	nd	19.16 ± 2.28
Procyanidin A	nd	0.12 ± 0.10	nd	0.19 ± 0.16	nd	0.44 ± 0.20	nd	0.75 ± 0.10	nd	1.27 ± 0.20
Procyanidin B	nd	2.45 ± 1.59	nd	12.10 ± 4.12	nd	6.89 ± 0.35	nd	16.78 ± 6.68	nd	13.33 ± 0.51
Procyanidin trimer	nd	nd	nd	0.27 ± 0.07	nd	0.21 ± 0.11	nd	0.41 ± 0.14	nd	0.42 ± 0.10
Propelargonidin dimer	nd	nd	nd	1.73 ± 0.44	nd	2.71 ± 1.24	nd	2.71 ± 1.45	nd	1.61 ± 0.15
Propelargonidin trimer	nd	nd	nd	0.79 ± 0.09	nd	0.98 ± 0.18	nd	1.22 ± 0.18	nd	0.85 ± 0.09
Total	nd	89.72 ± 11.54 [c]	0.02 ± 0.00 [d]	197.61 ± 31.34 [b]	0.05 ± 0.01 [d]	186.56 ± 6.13 [b]	0.02 ± 0.00 [d]	246.94 ± 38.98 [a]	0.02 ± 0.00 [d]	212.01 ± 14.05 [b]
**Stilbens (mg REE/100g DW)**										
Resveratrol	0.06 ± 0.00	nd	nd	nd	nd	nd	nd	nd	0.06 ± 0.00	nd
Total	0.06 ± 0.00 [a]	nd	nd	nd	nd	nd	nd	nd	0.06 ± 0.00 [a]	nd
**Cinnamic acids (mg CAE/100g DW)**										
Ferulic acid	nd	nd	nd	nd	nd	nd	0.11 ± 0.02	nd	nd	nd
Di-coumaroyl hexose	nd	nd	nd	nd	nd	nd	nd	nd	0.19 ± 0.02	nd
Total	nd	nd	nd	nd	nd	nd	0.11 ± 0.02 [b]	nd	0.19 ± 0.02 [a]	nd

[1] Values are expressed as means of triplicate determinations ± standard deviation (S.D.). nd, not detected. For the total concentrations, different small letters within row indicate significant differences (Duncan´s test, $p = 0.05$).

[2] Ellagic acid, (-)-epicatechin, gallic acid, kaempferol, myricetin, penta-*O*-galloyl-glucose, quercetin, resveratrol, and rutin are quantified by their standard, respectively, and expressed as mg/100g DW. GAE: gallic acid equivalent; RE: rutin equivalent; EE: (-)-epicatechin equivalent; REE: resveratrol equivalent; CAE: caffeic acid equivalent.
