# Supplementary material for: Profile of Polyphenol Compounds of Five Muscadine Grapes Cultivated in the United States and in Newly Adapted Locations in China
Source: Int J Mol Sci. 2017 Mar 14;18(3):631. doi: 10.3390/ijms18030631 (PMC5372644; doi:10.3390/ijms18030631)
Supplement: Supplementary file 1 [file ijms-18-00631-s001.zip › Table S2.pdf]

Table S2. Total and individual polyphenol concentrations identified in skins and seeds of five muscadine grapes from Nanning-Guangxi in the 2012 season.[1]

**Compounds** [2]	**Noble**		**Alachua**		**Carlos**		**Fry**		**Granny Val**	
	**skins**	**seeds**	**skins**	**seeds**	**skins**	**seeds**	**skins**	**seeds**	**skins**	**seeds**
**Ellagic acids and precursors (mg GAE/100g DW)**										
Ellagic acid derivatives										
Ellagic acid	124.67 ± 3.16	144.15 ± 7.71	137.19 ± 3.21	32.31 ± 1.51	100.2 ± 7.53	39.61 ± 5.81	117.42 ± 5.69	28.45 ± 6.73	144.3 ± 12.96	46.18 ± 11.38
Mono-*O*-methyl-ellagic acid	2.82 ± 0.15	10.20 ± 0.53	6.56 ± 0.82	4.63 ± 0.48	3.02 ± 0.07	4.58 ± 0.26	8.22 ± 0.21	3.09 ± 0.55	6.43 ± 0.71	4.95 ± 0.22
Mono-*O*-methyl-ellagic acid rhamnoside	0.71 ± 0.24	0.53 ± 0.00	nd	0.55 ± 0.02	nd	0.54 ± 0.00	nd	0.53 ± 0.00	nd	nd
Ducheside A	5.31 ± 1.68	7.11 ± 0.44	2.54 ± 1.33	0.92 ± 0.03	2.37 ± 0.83	2.08 ± 0.98	5.45 ± 0.52	1.26 ± 0.36	5.81 ± 2.69	1.11 ± 0.17
Ducheside B	2.75 ± 1.00	29.14 ± 1.89	1.91 ± 0.65	6.49 ± 0.87	2.17 ± 0.73	9.60 ± 1.62	8.45 ± 0.74	12.41 ± 2.05	7.33 ± 3.44	12.35 ± 1.61
Di-*O*-methyl-ellagic acid	0.41 ± 0.02	6.08 ± 0.26	0.94 ± 0.03	1.58 ± 0.11	0.46 ± 0.01	1.21 ± 0.04	1.21 ± 0.02	1.41 ± 0.05	0.75 ± 0.15	2.33 ± 0.06
Di-*O*-methyl-ellagic acid 4-*O*-rhamnoside	nd	0.56 ± 0.01	nd	0.54 ± 0.01	nd	nd	nd	nd	nd	nd
Tri-*O*-methyl-ellagic acid	5.40 ± 0.22	4.81 ± 0.81	10.70 ± 0.66	4.96 ± 4.53	4.26 ± 0.25	2.20 ± 0.30	14.04 ± 1.69	2.34 ± 0.27	8.30 ± 1.97	3.48 ± 1.28
Galloyl-tri-*O*-methyl-ellagic acid hexoside	nd	0.79 ± 0.02	nd	nd	nd	nd	nd	0.82 ± 0.00	nd	nd
Ellagic acid glucoside	9.73 ± 0.34	5.52 ± 0.29	9.84 ± 1.25	1.79 ± 0.10	11.89 ± 2.03	2.15 ± 0.36	9.84 ± 1.10	2.74 ± 0.40	18.92 ± 4.65	3.61 ± 0.35
Ellagic acid-coumaroyl hexoside	0.19 ± 0.03	nd	nd	nd	0.13 ± 0.02	nd	nd	nd	nd	nd
Ellagic acid-dihexoside	0.20 ± 0.02	nd	0.26 ± 0.03	nd	0.29 ± 0.12	nd	0.58 ± 0.13	nd	0.29 ± 0.15	nd
Ellagic acid derivative m/z 447	nd	nd	nd	nd	nd	nd	0.55 ± 0.11	nd	0.48 ± 0.18	nd
Ellagitannins										
Ellagitannin m/z 443	nd	0.85 ± 0.04	nd	1.33 ± 0.09	0.25 ± 0.06	1.05 ± 0.13	0.28 ± 0.03	0.98 ± 0.13	0.42 ± 0.18	1.06 ± 0.16
Phloretin	nd	0.96 ± 0.11	nd	1.56 ± 0.08	nd	1.41 ± 0.07	nd	1.12 ± 0.36	0.11 ± 0.00	1.15 ± 0.22
Methyl brevifolin carboxylate	24.91 ± 2.32	12.57 ± 1.00	27.12 ± 7.26	2.49 ± 0.69	29.07 ±5.31	4.33 ± 0.91	16.59 ± 5.52	8.43 ± 2.23	25.89 ± 6.85	7.08 ± 2.90
Valoneic acid dilactone	0.73 ± 0.04	nd	0.78 ± 0.09	nd	0.67 ± 0.16	nd	0.54 ± 0.05	nd	1.10 ± 0.29	nd
HHDP glucose	0.52 ± 0.15	4.19 ± 0.14	0.44 ± 0.20	nd	0.28 ± 0.01	0.91 ± 0.14	0.36 ± 0.12	1.38 ± 0.10	0.75 ± 0.42	nd
Ellagitannin m/z 497	0.81 ± 0.33	nd	nd	nd	0.61 ± 0.16	nd	1.36 ± 0.57	nd	1.36 ± 0.51	nd
Gallagic acid	0.54 ± 0.05	nd	0.67 ± 0.06	nd	0.40 ± 0.02	nd	0.57 ± 0.07	nd	0.73 ± 0.11	nd
Ellagitannin m/z 603	3.92 ± 0.12	nd	4.15 ± 0.02	nd	3.84 ± 0.51	nd	4.15 ± 0.10	nd	5.63 ± 1.01	0.53 ± 0.00
Galloyl-HHDP-glucose (Corilagin, Strictinin)	nd	1.90 ± 0.08	0.18 ± 0.09	0.63 ± 0.09	nd	0.60 ± 0.23	0.20 ± 0.14	1.13 ± 0.08	0.26 ± 0.17	0.90 ± 0.25
HHDP-galloyl-glucose (Isostrictinin)	nd	1.55 ± 0.65	0.13 ± 0.01	0.58 ± 0.02	0.11 ± 0.00	0.68 ± 0.01	0.12 ± 0.02	1.00 ± 0.18	0.22 ± 0.10	0.75 ± 0.05
Ellagitannin m/z 633	0.41 ± 0.07	nd	0.41 ± 0.15	nd	0.27 ± 0.20	nd	0.23 ± 0.17	nd	0.19 ± 0.07	nd
Ellagitannin m/z 643	nd	nd	4.75 ± 0.93	nd	nd	nd	nd	nd	nd	nd
Ellagitannin m/z 671	nd	nd	0.71 ± 0.11	nd	0.12 ± 0.01	nd	nd	nd	nd	nd
Ellagitannin m/z 681	0.78 ± 0.12	nd	0.46 ± 0.07	nd	nd	nd	nd	nd	nd	nd
Ellagitannin m/z 689	0.77 ± 0.10	nd	0.46 ± 0.11	nd	nd	nd	nd	nd	nd	nd
Ellagitannin m/z 721	0.13 ± 0.01	nd	0.11 ± 0.01	nd	nd	nd	nd	nd	nd	nd
HHDP-galloyl-glucose (Isostrictinin)	nd	1.55 ± 0.65	0.13 ± 0.01	0.58 ± 0.02	0.11 ± 0.00	0.68 ± 0.01	0.12 ± 0.02	1.00 ± 0.18	0.22 ± 0.10	0.75 ± 0.05

Supplementary Table 2 *Cont.*

Compounds [2]	Noble		Alachua		Carlos		Fry		Granny Val	
	skins	seeds	skins	seeds	skins	seeds	skins	seeds	skins	seeds
Ellagitannin m/z 779	0.38 ± 0.04	nd	0.38 ± 0.09	nd	0.29 ± 0.06	nd	0.63 ± 0.03	nd	0.77 ± 0.29	nd
Pedunculagin α/ß isomer (Di-HHDP-glucose)	nd	0.81 ± 0.02	nd	nd	nd	0.53 ± 0.00	nd	nd	nd	nd
Tellimagrandin I (Digalloyl-HHDP-glucose)	nd	7.43 ± 0.76	0.14 ± 0.01	1.23 ± 0.15	nd	1.27 ± 0.12	nd	2.41 ± 0.29	nd	1.68 ± 0.31
Ellagitannin m/z 797	nd	nd	0.17 ± 0.04	nd	nd	nd	0.40 ± 0.08	nd	0.39 ± 0.17	nd
Isomallotinic acid	nd	0.74 ± 0.02	nd	nd	nd	0.89 ± 0.01	nd	nd	nd	nd
Ellagitannin m/z 831	nd	nd	nd	nd	0.28 ± 0.03	nd	0.73 ± 0.05	nd	0.68 ± 0.31	nd
Ellagitannin m/z 861	0.20 ± 0.01	nd	0.18 ± 0.08	nd	nd	nd	0.28 ± 0.01	nd	0.34 ± 0.11	nd
Galloyl-bis-HHDP-glucose (Casuarinin)	nd	0.95 ± 0.02	nd	nd	nd	0.62 ± 0.02	nd	0.59 ± 0.00	nd	nd
Tellimagrandin II (Trigalloyl-HHDP-glucose)	nd	1.41 ± 0.21	nd	0.60 ± 0.01	nd	0.60 ± 0.00	nd	1.13 ± 0.09	nd	0.79 ± 0.14
Chebulagic acid	nd	nd	nd	nd	nd	0.66 ± 0.07	nd	nd	nd	nd
Ellagitannin m/z 957	0.18 ± 0.02	nd	0.24 ± 0.03	nd	0.22 ± 0.04	nd	0.24 ± 0.05	nd	0.31 ± 0.05	nd
Ellagitannin m/z 967	nd	0.69 ± 0.05	nd	0.71 ± 0.08	nd	0.73 ± 0.06	nd	nd	nd	0.66 ± 0.04
Precursors (Gallic acid derivatives)										
Gallic acid	3.09 ± 0.58	46.24 ± 7.22	2.48 ± 0.74	48.98 ± 7.67	1.64 ± 0.61	46.45 ± 10.91	1.65 ± 0.22	35.36 ± 2.55	3.64 ± 1.38	34.89 ± 4.04
Methyl gallate	6.38 ± 0.39	16.39 ± 8.10	5.73 ± 1.28	10.27 ± 2.14	5.91 ± 3.52	51.85 ± 7.42	6.13 ± 2.05	40.09 ± 12.69	10.44 ± 2.31	4.04 ± 3.29
Di-*O*-galloyl quinic acid	0.39 ± 0.04	nd	0.25 ± 0.14	nd	0.22 ± 0.03	nd	0.16 ± 0.02	nd	1.26 ± 1.08	nd
Mono-*O*-galloyl-glucose	0.43 ± 0.13	6.35 ± 0.88	0.57 ± 0.19	4.31 ± 0.55	0.31 ± 0.12	4.06 ± 1.01	0.28 ± 0.03	9.07 ± 2.63	0.72 ± 0.32	7.79 ± 4.31
Di-*O*-galloyl-glucose	0.51 ± 0.14	0.67 ± 0.02	0.54 ± 0.29	0.76 ± 0.14	0.50 ± 0.20	0.64 ± 0.03	0.52 ± 0.02	0.69 ± 0.02	0.75 ± 0.15	0.69 ± 0.05
Tri-*O*-galloyl-glucose	nd	4.90 ± 0.93	nd	5.48 ± 0.08	0.11 ± 0.00	2.57 ± 0.15	0.07 ± 0.06	4.39 ± 2.19	0.16 ± 0.09	3.57 ± 1.20
Tetra-*O*-galloyl-glucose	nd	15.41 ± 1.61	nd	26.86 ± 1.72	nd	14.68 ± 0.57	nd	17.73 ± 0.96	nd	12.00 ± 0.70
Penta-*O*-galloyl-glucose	nd	234.88 ± 24.39	nd	418.34 ± 26.94	nd	209.93 ± 12.71	nd	307.42 ± 56.71	nd	246.49 ± 41.84
Total	197.25 ± 4.34 ^{d,e}	567.76 ± 38.01 ^{a}	220.99 ± 6.92 ^{d,e}	577.89 ± 27.30 ^{a}	169.89 ± 13.49 ^{e}	406.42 ± 24.46 ^{c}	201.26 ± 2.27 ^{d,e}	486.00 ± 58.05 ^{b}	248.73 ± 22.72 ^{d}	398.08 ± 49.60 ^{c}
**Flavonols (mg RE/100g DW)**										
Myricetin	0.40 ± 0.27	nd	0.14 ± 0.08	nd	nd	nd	nd	nd	nd	nd
Myricetin tri-methyl ether	9.81 ± 1.97	nd	8.86 ± 1.59	nd	13.81 ± 1.71	nd	30.81 ± 4.16	nd	24.18 ± 6.12	0.30 ± 0.10
Myricetin 3-*O*-glucuronide	0.54 ± 0.17	nd	0.43 ± 0.21	nd	0.35 ± 0.16	nd	nd	nd	1.42 ± 0.31	nd
Myricetin 3-*O*-glucoside	0.51 ± 0.13	nd	1.03 ± 1.02	nd	nd	nd	nd	nd	0.15 ± 0.03	nd
Myricetin 3,4'-diglucoside	0.22 ± 0.02	nd	0.24 ± 0.07	nd	nd	nd	nd	nd	nd	nd
Quercetin	0.96 ± 0.47	nd	1.82 ± 0.71	nd	0.24 ± 0.11	nd	0.70 ± 0.61	nd	1.99 ± 1.90	nd
Quercetin 3-glucuronide	0.33 ± 0.11	nd	0.43 ± 0.02	nd	nd	nd	nd	nd	0.34 ± 0.05	nd
Quercetin 3-*O*-glucoside	1.16 ± 0.12	0.14 ± 0.03	1.27 ± 0.05	0.12 ± 0.02	nd	0.12 ± 0.03	nd	0.10 ± 0.01	nd	nd
Quercetin malonyl glucoside	nd	nd	nd	nd	nd	0.08 ± 0.00	nd	nd	nd	nd
Rutin	0.11 ± 0.02	nd	nd	nd	nd	nd	nd	nd	nd	nd
Quercetin 3,4'-diglucoside	0.78 ± 0.07	nd	0.78 ± 0.09	nd	nd	nd	nd	nd	nd	nd

Supplementary Table 2 *Cont.*

Compounds [2]	Noble		Alachua		Carlos		Fry		Granny Val	
	skins	seeds	skins	seeds	skins	seeds	skins	seeds	skins	seeds
Dihydroquercetin	nd	2.93 ± 0.86	nd	4.74 ± 0.27	nd	6.26 ± 1.19	nd	3.79 ± 2.59	nd	2.96 ± 1.81
Dihydroquercetin 3-*O*-hexoside	0.78 ± 0.15	nd	0.70 ± 0.10	nd	1.02 ± 0.12	nd	0.42 ± 0.17	nd	1.48 ± 0.54	nd
Dihydroquercetin caffeoyl glucoside	4.55 ± 0.57	nd	1.02 ± 0.15	nd	nd	nd	nd	nd	nd	nd
Kaempferol	0.21 ± 0.15	nd	0.15 ± 0.11	nd	nd	nd	0.28 ± 0.10	nd	nd	nd
Kaempferol 3-*O*-rhamnoside	0.79 ± 0.14	nd	1.86 ± 0.32	0.10 ± 0.01	2.62 ± 1.17	nd	2.18 ± 0.80	nd	2.19 ± 0.79	nd
Kaempferol 3-*O*-glucoside	1.14 ± 0.25	2.43 ± 0.71	0.16 ± 0.01	1.91 ± 0.30	nd	0.83 ± 0.34	0.57 ± 0.05	1.18 ± 0.50	nd	1.59 ± 0.83
Amurensin	nd	nd	nd	nd	0.38 ± 0.08	nd	0.20 ± 0.06	nd	0.33 ± 0.06	nd
Kaempferol acetyl glucoside	nd	nd	1.02 ± 0.25	nd	0.85 ± 0.11	nd	nd	nd	nd	0.09 ± 0.01
Kaempferol dihexose	0.69 ± 0.03	nd	nd	nd	nd	nd	nd	nd	nd	nd
Kaempferol 3-*O*-rutinoside	nd	nd	0.36 ± 0.13	nd	0.52 ± 0.13	nd	0.40 ± 0.12	nd	0.32 ± 0.18	nd
Dihydrokaempferol 3-O-arabinoside	0.36 ± 0.01	0.10 ± 0.01	0.19 ± 0.07	0.11 ± 0.02	0.11 ± 0.00	nd	0.08 ± 0.00	nd	0.08 ± 0.00	0.09 ± 0.00
Dihydrokaempferol 7-*O*-glucoside	nd	nd	nd	0.99 ± 0.05	nd	1.44 ± 0.18	nd	1.19 ± 0.05	nd	3.23 ± 0.35
Isorhamnetin	nd	1.20 ± 0.59	nd	1.45 ± 0.18	nd	1.70 ± 0.30	nd	1.54 ± 1.11	nd	1.35 ± 0.92
Isorhamnetin 3-glucuronide	nd	nd	0.08 ± 0.01	nd	0.15 ± 0.04	nd	0.33 ± 0.01	nd	0.28 ± 0.03	nd
Isorhamnetin 3-glucoside	nd	0.14 ± 0.03	0.61 ± 0.06	nd	nd	nd	nd	0.19 ± 0.07	nd	0.20 ± 0.06
Laricitrin	0.10 ± 0.01	nd	0.12 ± 0.02	nd	0.11 ± 0.04	nd	0.43 ± 0.07	nd	0.13 ± 0.06	nd
Laricitrin 3-*O*-glucoside	1.20 ± 0.24	0.08 ± 0.01	1.05 ± 0.18	0.09 ± 0.01	nd	0.08 ± 0.00	0.09 ± 0.01	nd	nd	nd
Syringetin	0.47 ± 0.14	nd	2.23 ± 2.27	nd	0.64 ± 0.33	nd	2.41 ± 1.80	nd	1.02 ± 0.36	nd
Syringetin 3-glucoside	1.16 ± 0.08	nd	nd	nd	nd	nd	nd	nd	nd	nd
Total	26.28 ± 3.94 [b]	7.03 ± 0.96 [c]	24.54 ± 4.66 [b]	9.5 ± 0.16 [c]	20.79 ± 2.97 [b]	10.52 ± 1.21 [c]	38.92 ± 5.11 [a]	7.98 ± 3.35 [c]	33.9 ± 6.10 [a]	9.82 ± 1.75 [c]
**Benzoic acids (mg GAE/100g DW)**										
Vanillic acid	0.16 ± 0.05	nd	0.11 ± 0.00	nd	nd	nd	nd	nd	0.11 ± 0.00	nd
Vanillic acid 4-glucoside	0.83 ± 0.40	0.57 ± 0.03	0.27 ± 0.08	0.58 ± 0.03	nd	nd	nd	0.55 ± 0.01	nd	0.57 ± 0.02
Syringic acid	0.18 ± 0.02	1.97 ± 0.06	0.15 ± 0.01	1.09 ± 0.03	nd	3.26 ± 0.25	nd	2.08 ± 0.06	0.12 ± 0.01	2.72 ± 0.18
Mono-hydroxybenzoic acid	0.18 ± 0.03	nd	0.18 ± 0.00	0.53 ± 0.00	0.20 ± 0.01	0.53 ± 0.00	0.21 ± 0.00	0.53 ± 0.00	0.18 ± 0.01	0.53 ± 0.00
Di-hydroxybenzoic acid	0.90 ± 0.05	0.60 ± 0.01	0.70 ± 0.02	0.58 ± 0.01	0.70 ± 0.06	0.60 ± 0.01	0.80 ± 0.18	0.57 ± 0.01	0.89 ± 0.37	0.62 ± 0.04
Di-hydroxybenzate ethyl ester	nd	nd	0.12 ± 0.00	nd	nd	nd	nd	nd	nd	nd
Brevifolin carboxylic acid	20.48 ± 1.21	0.73 ± 0.17	nd	0.64 ± 0.07	17.72 ±5.18	0.57 ± 0.02	15.94 ± 1.10	0.64 ± 0.07	22.73 ± 6.92	0.62 ± 0.05
Mucic acid digallate	0.37 ± 0.08	nd	0.55 ± 0.32	nd	0.49 ± 0.11	nd	0.50 ± 0.39	nd	0.67 ± 0.09	nd
Mucic acid lactone digallate	0.37 ± 0.06	nd	0.61 ± 0.44	nd	0.39 ± 0.10	0.54 ± 0.01	0.52 ± 0.31	nd	0.76 ± 0.26	nd
Mucic acid methyl ester gallate	12.40 ± 1.72	nd	4.40 ± 2.64	2.07 ± 0.22	1.10 ± 0.28	nd	3.52 ± 0.26	nd	7.23 ± 6.66	nd
Mucic acid methyl ester digallate	0.39 ± 0.07	0.55 ± 0.01	0.42 ± 0.13	nd	0.31 ± 0.08	nd	0.16 ± 0.05	nd	0.49 ± 0.23	nd
Citric acid	nd	13.74 ±5.71	nd	12.53 ± 4.02	0.13 ± 0.01	0.69 ± 0.16	nd	1.13 ± 0.76	nd	13.15 ± 1.89
1-*O*-protocatechuoyl-glucose	0.35 ± 0.07	0.94 ± 0.03	nd	1.09 ± 0.03	0.39 ± 0.18	1.03 ± 0.07	0.71 ± 0.05	1.12 ± 0.15	0.79 ± 0.3	1.46 ± 0.11
Total	36.6 ± 1.61 [a]	19.1 ± 5.58 [b]	7.51 ± 2.75 [c]	19.11 ± 4.02 [b]	21.42 ± 5.41 [b]	7.22 ± 0.48 [c]	22.37 ± 0.73 [b]	6.62 ± 0.92 [c]	33.97 ± 8.38 [a]	19.65 ± 1.93 [b]
**Flavan-3-ols (mg EE/100g DW)**										
(Epi)catechin	0.03 ± 0.00	78.91 ± 7.00	0.02 ± 0.00	156.6 ± 10.15	0.02 ± 0.00	166.63 ± 11.34	0.02 ± 0.00	100.37 ± 20.38	0.02 ± 0.00	112.43 ± 28.92
(Epi)catechin gallate	nd	17.34 ± 1.12	nd	38.49 ± 7.98	nd	32.95 ± 5.70	0.02 ± 0.00	25.19 ± 1.67	nd	27.10 ± 2.29
(Epi)catechin hexoside	0.03 ± 0.00	1.61 ± 0.16	nd	6.46 ± 0.67	0.03 ± 0.01	4.97 ± 0.92	nd	2.09 ± 0.50	nd	2.02 ± 0.10

Supplementary Table 2 ***Cont.***

Compounds [2]	Noble		Alachua		Carlos		Fry		Granny Val	
	skins	seeds	skins	seeds	skins	seeds	skins	seeds	skins	seeds
(Epi)gallocatechin	0.02 ± 0.00	1.26 ± 0.40	nd	1.98 ± 0.26	nd	0.92 ± 0.15	nd	1.46 ± 0.30	nd	1.43 ± 0.54
(Epi)gallocatechin gallate	nd	1.29 ± 0.38	nd	2.40 ± 0.83	nd	3.20 ± 1.10	0.02 ± 0.00	1.48 ± 0.40	nd	1.42 ± 0.09
Methyl epigallocatechin -3-gallate	nd	21.91 ± 10.23	0.03 ± 0.01	19.41 ± 4.48	nd	25.47 ± 0.52	nd	22.13 ± 7.91	0.02 ± 0.00	16.51 ± 7.10
Procyanidin A	nd	0.27 ± 0.24	nd	0.09 ± 0.09	nd	1.07 ± 0.23	nd	0.60 ± 0.50	nd	0.69 ± 1.02
Procyanidin B	nd	5.82 ± 0.77	nd	24.48 ± 2.11	nd	15.01 ± 1.17	nd	14.05 ± 7.51	nd	9.65 ± 4.37
Procyanidin trimer	nd	0.03 ± 0.03	nd	0.46 ± 0.23	0.02 ± 0.00	0.42 ± 0.17	nd	0.44 ± 0.45	nd	0.21 ± 0.09
Propelargonidin dimer	nd	nd	nd	0.59 ± 0.02	nd	nd	nd	nd	nd	1.09 ± 0.22
Total	0.08 ± 0.00 [d]	128.43 ± 12.16 [c]	0.05 ± 0.01 [d]	250.96 ± 21.35 [a]	0.08 ± 0.01 [d]	250.65 ± 16.96 [a]	0.07 ± 0.00 [d]	167.81 ± 21.66 [b]	0.04 ± 0.00 [d]	172.55 ± 33.17 [b]
**Stilbens (mg REE/100g DW)**										
Resveratrol	nd	nd	0.06 ± 0.00	nd	0.06 ± 0.00	nd	nd	nd	0.06 ± 0.00	nd
Resveratrol 3-*O*-glucoside	nd	nd	nd	nd	0.12 ± 0.01	nd	nd	nd	nd	nd
Total	nd	nd	0.06 ± 0.00[b]	nd	0.18 ± 0.01[a]	nd	nd	nd	0.06 ± 0.00[b]	nd
**Cinnamic acids (mg CAE/100g DW)**										
Di-coumaroyl hexose	nd	nd	nd	nd	0.14 ± 0.07	nd	nd	nd	nd	nd
Total	nd	nd	nd	nd	0.14 ± 0.07[a]	nd	nd	nd	nd	nd

[1] Values are expressed as means of triplicate determinations ± S.D. nd, not detected. For the total concentrations, different small letters within row indicate significant differences (Duncan´s test, $p = 0.05$).

[2] Ellagic acid, (-)-epicatechin, gallic acid, kaempferol, myricetin, penta-*O*-galloyl-glucose, quercetin, resveratrol, and rutin are quantified by their standard, respectively, and expressed as mg/100g DW. GAE: gallic acid equivalent; RE: rutin equivalent; EE: (-)-epicatechin equivalent; REE: resveratrol equivalent; CAE: caffeic acid equivalent.
