# Supplementary material for: Profile of Polyphenol Compounds of Five Muscadine Grapes Cultivated in the United States and in Newly Adapted Locations in China
Source: Int J Mol Sci. 2017 Mar 14;18(3):631. doi: 10.3390/ijms18030631 (PMC5372644; doi:10.3390/ijms18030631)
Supplement: Supplementary file 1 [file ijms-18-00631-s001.zip › Table S3.pdf]

Table S3. Total and individual polyphenol concentrations identified in skins and seeds of three muscadine grapes from Pu'er-Yunnan in the 2012 season.[1]

Compounds [2]	Noble		Carlos		Granny Val	
	skins	seeds	skins	seeds	skins	seeds
**Ellagic acids and precursors (mg GAE/100g DW)**						
Ellagic acid derivatives						
Ellagic acid	109.68 ± 16.73	110.36 ± 8.55	100.78 ± 1.47	55.00 ± 4.11	110.42 ± 14.3	45.24 ± 9.85
Mono-*O*-methyl-ellagic acid	1.57 ± 0.41	6.44 ± 0.81	2.48 ± 0.06	6.37 ± 1.09	6.09 ± 2.39	5.01 ± 0.37
Mono-*O*-methyl-ellagic acid rhamnoside	0.27 ± 0.12	0.53 ± 0.00	nd	nd	nd	0.53 ± 0.00
Mono-*O*-methyl-ellagic acid deoxyhexoside	0.12 ± 0.01	nd	nd	nd	0.11 ± 0.00	nd
Ducheside A	6.90 ± 1.58	8.68 ± 2.54	10.05 ± 1.68	6.38 ± 1.93	14.83 ± 1.74	12.22 ± 1.44
Ducheside B	13.95 ± 1.45	2.68 ± 0.69	7.84 ± 1.40	0.91 ± 0.11	11.03 ± 4.58	1.22 ± 0.29
Di-*O*-methyl-ellagic acid	0.34 ± 0.04	4.43 ± 0.29	0.29 ± 0.02	1.62 ± 0.17	0.90 ± 0.25	2.17 ± 0.18
Di-*O*-methyl-ellagic acid 4-*O*-rhamnoside	nd	nd	nd	nd	0.11 ± 0.00	nd
Tri-*O*-methyl-ellagic acid	4.40 ± 0.92	10.38 ± 4.91	2.65 ± 0.25	9.72 ± 1.88	8.46 ± 2.05	5.51 ± 0.61
Ellagic acid glucoside	6.81 ± 1.69	2.33 ± 0.11	7.23 ± 0.63	2.46 ± 0.53	11.39 ± 3.81	3.83 ± 0.43
Ellagic acid coumaroyl hexoside	0.35 ± 0.08	nd	0.15 ± 0.01	nd	0.25 ± 0.08	nd
Ellagic acid dihexoside	0.22 ± 0.02	nd	nd	nd	0.21 ± 0.07	nd
Ellagic acid derivative m/z 447	1.26 ± 0.33	nd	0.65 ± 0.04	nd	nd	nd
Ellagitannins						
Ellagitannin m/z 443	0.26 ± 0.03	0.71 ± 0.06	0.70 ± 0.07	1.08 ± 0.21	0.62 ± 0.22	1.03 ± 0.09
Phloretin	nd	1.56 ± 0.05	nd	2.08 ± 0.23	nd	1.10 ± 0.10
Methyl brevifolin carboxylate	6.81 ± 1.07	11.08 ± 1.31	5.17 ± 1.27	4.13 ± 1.01	5.22 ± 3.50	7.70 ± 0.46
Valoneic acid dilactone	0.32 ± 0.10	nd	0.35 ± 0.03	nd	0.49 ± 0.13	nd
HHDP glucose	1.12 ± 0.19	2.96 ± 0.34	1.21 ± 0.13	0.79 ± 0.06	1.62 ± 0.39	1.20 ± 0.07
Ellagitannin m/z 497	0.26 ± 0.19	nd	1.75 ± 0.14	nd	1.33 ± 0.45	nd
Eriodictyol coumaroyl hexose	nd	nd	nd	0.63 ± 0.07	nd	1.28 ± 0.14
Gallagic acid	0.43 ± 0.01	nd	0.36 ± 0.02	nd	0.52 ± 0.15	nd
Ellagitannin m/z 603	nd	nd	nd	nd	5.08 ± 1.29	nd
Galloyl-HHDP-glucose (Corilagin, Strictinin)	0.21 ± 0.04	2.10 ± 0.54	0.19 ± 0.07	0.68 ± 0.08	0.32 ± 0.18	0.85 ± 0.02
HHDP-galloyl-glucose (Isostrictinin)	0.24 ± 0.03	1.41 ± 0.38	0.23 ± 0.01	0.61 ± 0.08	0.28 ± 0.17	0.73 ± 0.14
Ellagitannin m/z 633	0.26 ± 0.21	nd	0.14 ± 0.04	nd	0.23 ± 0.14	nd
Ellagitannin m/z 643	21.58 ± 1.04	nd	0.22 ± 0.01	nd	0.35 ± 0.18	nd
Ellagitannin m/z 671	2.53 ± 0.24	nd	0.12 ± 0.01	nd	nd	nd
Ellagitannin m/z 681	2.60 ± 0.87	nd	nd	nd	nd	nd
Ellagitannin m/z 689	2.21 ± 0.63	nd	nd	nd	nd	nd
Ellagitannin m/z 721	0.20 ± 0.04	nd	nd	nd	nd	nd
Ellagitannin m/z 779	nd	nd	1.26 ± 0.40	nd	1.81 ± 0.84	nd
Punicalin α/ß isomer	nd	nd	nd	nd	0.32 ± 0.11	nd
Pedunculagin α/ß isomer (Di-HHDP-glucose)	nd	nd	nd	0.53 ± 0.01	nd	0.56 ± 0.02
Tellimagrandin I (Digalloyl-HHDP-glucose)	0.17 ± 0.01	3.38 ± 0.58	nd	1.57 ± 0.27	0.22 ± 0.09	1.70 ± 0.17
Ellagitannin m/z 797	0.42 ± 0.02	nd	0.53 ± 0.01	nd	0.74 ± 0.25	nd
Isomallotinic acid	0.12 ± 0.01	0.8 ± 0.08	nd	1.04 ± 0.15	0.17 ± 0.05	0.9 ± 0.03
Ellagitannin m/z 831	1.58 ± 0.28	nd	nd	nd	3.36 ± 2.13	nd
Ellagitannin m/z 861	0.49 ± 0.08	nd	0.54 ± 0.10	nd	0.65 ± 0.11	nd
Galloyl-bis-HHDP-glucose (Casuarinin)	nd	0.83 ± 0.05	nd	0.71 ± 0.05	0.10 ± 0.00	0.76 ± 0.04
Tellimagrandin II (Trigalloyl-HHDP-glucose)	nd	1.16 ± 0.09	nd	0.64 ± 0.03	nd	0.71 ± 0.06
Chebulagic acid	nd	nd	nd	0.76 ± 0.14	nd	0.75 ± 0.05
Ellagitannin m/z 957	0.27 ± 0.00	nd	0.29 ± 0.01	nd	0.23 ± 0.11	nd
Ellagitannin m/z 967	nd	0.67 ± 0.05	n	0.88 ± 0.12	nd	0.79 ± 0.02
Total	196.85 ± 17.01 [b]	380.61 ± 28.78 [a]	155.07 ± 3.92 [c]	381.49 ± 20.34 [a]	201.41 ± 26.46 [b]	224.19 ± 7.81 [b]
Precursors (Gallic acid derivatives)						
Gallic acid	3.76 ± 0.34	48.19 ± 5.81	4.24 ± 0.22	52.83 ± 6.24	5.38 ± 3.31	nd
Methyl gallate	2.90 ± 0.78	65.67 ± 8.34	3.07 ± 0.95	56.19 ± 13.3	4.92 ± 3.78	6.86 ± 2.60
Di-*O*-galloyl-quinic acid	0.22 ± 0.07	nd	0.36 ± 0.13	nd	1.16 ± 0.98	nd
Mono-*O*-galloyl-glucose	0.64 ± 0.15	3.51 ± 2.23	0.56 ± 0.10	3.41 ± 2.00	0.72 ± 0.21	4.97 ± 3.20
Di-*O*-galloyl-glucose	0.66 ± 0.14	0.64 ± 0.06	1.22 ± 0.23	0.91 ± 0.56	1.47 ± 0.50	0.62 ± 0.00
Tri-*O*-galloyl-glucose	nd	2.03 ± 0.62	0.11 ± 0.00	2.04 ± 0.54	0.11 ± 0.01	2.95 ± 0.63

Supplementary Table 3 ***Cont.***

Compounds [2]	Noble		Carlos		Granny Val	
	skins	seeds	skins	seeds	skins	seeds
Tetra-*O*-galloyl-glucose	nd	8.01 ± 1.41	nd	12.07 ± 3.26	nd	7.63 ± 0.52
Penta-*O*-galloyl-glucose	nd	80.08 ± 11.42	nd	155.46 ± 8.06	nd	105.34 ± 7.37
Gallic acid derivative m/z 569	0.16 ± 0.00	nd	0.33 ± 0.05	nd	0.21 ± 0.08	nd
Gallotannin m/z 803	0.57 ± 0.07	nd	nd	nd	nd	nd
**Flavonols (mg RE/100g DW)**						
Myricetin	7.81 ± 1.68	nd	nd	nd	nd	nd
Myricetin tri-methyl ether	nd	nd	16.61 ± 2.76	nd	14.35 ± 2.89	0.44 ± 0.12
Myricetin 3-*O*-glucuronide	nd	nd	0.23 ± 0.02	nd	nd	nd
Myricetin 3-*O*-glucoside	2.54 ± 0.07	nd	0.13 ± 0.01	nd	0.16 ± 0.04	nd
Myricetin 3,4'-diglucoside	0.79 ± 0.12	nd	nd	nd	nd	nd
Quercetin	3.50 ± 0.58	nd	1.47 ± 0.69	nd	0.47 ± 0.27	nd
Quercetin 3-glucuronide	nd	nd	0.17 ± 0.02	nd	0.27 ± 0.09	nd
Quercetin 3-*O*-glucoside	1.15 ± 0.33	nd	nd	nd	nd	0.13 ± 0.00
Quercetin malonyl glucoside	0.09 ± 0.01	nd	nd	nd	nd	0.08 ± 0.00
Rutin	0.08 ± 0.00	nd	nd	nd	nd	nd
Quercetin 3,4'-diglucoside	2.30 ± 0.34	nd	nd	nd	nd	nd
Dihydroquercetin	nd	5.79 ± 1.32	nd	9.64 ± 1.76	nd	2.06 ± 1.03
Dihydroquercetin 3-*O*-hexoside	0.46 ± 0.15	nd	nd	nd	0.95 ± 0.46	nd
Dihydroquercetin caffeoyl glucoside	5.00 ± 0.26	nd	nd	nd	0.13 ± 0.05	nd
Kaempferol	0.65 ± 0.22	nd	nd	nd	nd	nd
Kaempferol 3-*O*-glucuronide	0.15 ± 0.01	nd	nd	nd	0.23 ± 0.08	nd
Kaempferol 3-*O*-rhamnoside	1.17 ± 0.17	nd	1.89 ± 0.38	nd	2.43 ± 1.57	nd
Kaempferol 3-*O*-glucoside	nd	0.53 ± 0.44	nd	nd	nd	2.43 ± 1.06
Kaempferol dihexose	0.69 ± 0.05	nd	nd	nd	nd	nd
Dihydrokaempferol 3-O-arabinoside	0.50 ± 0.11	nd	0.08 ± 0.00	0.10 ± 0.00	0.10 ± 0.02	0.09 ± 0.01
Dihydrokaempferol 7-*O*-glucoside	nd	1.29 ± 0.16	nd	2.03 ± 0.41	nd	4.86 ± 0.68
Isorhamnetin	0.40 ± 0.06	2.88 ± 0.40	nd	2.62 ± 0.44	0.14 ± 0.05	1.65 ± 0.99
Isorhamnetin 3-glucuronide	0.09 ± 0.01	nd	0.22 ± 0.01	0.29 ± 0.09	0.24 ± 0.14	nd
Isorhamnetin 3-glucoside	0.83 ± 0.35	nd	nd	nd	nd	0.29 ± 0.13
Isorhamnetin sophorose	0.92 ± 0.07	nd	0.08 ± 0.00	nd	0.09 ± 0.01	nd
Laricitrin	0.17 ± 0.02	nd	0.09 ± 0.01	nd	nd	nd
Laricitrin 3-*O*-glucoside	2.21 ± 0.29	nd	nd	nd	nd	0.09 ± 0.00
Syringetin	7.39 ± 1.01	nd	12.13 ± 5.32	nd	16.13 ± 4.35	nd
Total	38.89 ± 1.73 [a]	10.48 ± 1.20 [c]	33.10 ± 3.74 [b]	14.67 ± 2.62 [c]	35.69 ± 5.50 [ab]	12.13 ± 0.46 [c]
**Benzoic acids (mg GAE/100g DW)**						
Vanillic acid	0.42 ± 0.10	nd	nd	nd	0.11 ± 0.00	nd
Vanillic acid 4-glucoside	3.93 ± 1.96	nd	0.15 ± 0.02	nd	nd	0.57 ± 0.02
Syringic acid	0.22 ± 0.01	4.82 ± 0.43	nd	2.70 ± 0.41	2.18 ± 0.91	2.50 ± 0.20
Mono-hydroxybenzoic acid	0.27 ± 0.03	0.53 ± 0.00	0.20 ± 0.01	0.53 ± 0.00	0.21 ± 0.04	0.53 ± 0.00
Di-hydroxybenzoic acid	0.49 ± 0.36	0.58 ± 0.03	0.42 ± 0.03	0.70 ± 0.09	0.44 ± 0.17	0.60 ± 0.01
Di-hydroxybenzate ethyl ester	nd	nd	nd	nd	0.35 ± 0.22	nd
Brevifolin carboxylic acid	10.43 ± 3.71	0.56 ± 0.02	3.54 ± 0.79	0.57 ± 0.02	5.02 ± 1.88	0.61 ± 0.06
Mucic acid digallate	0.61 ± 0.28	nd	0.83 ± 0.16	nd	1.23 ± 0.32	nd
Mucic acid lactone digallate	0.62 ± 0.25	nd	0.76 ± 0.06	nd	0.90 ± 0.35	nd
Mucic acid methyl ester gallate	nd	nd	nd	nd	nd	1.32 ± 0.59
Mucic acid methyl ester digallate	0.62 ± 0.17	nd	0.44 ± 0.04	nd	0.82 ± 0.30	nd
Citric acid	0.25 ± 0.04	0.68 ± 0.17	0.24 ± 0.02	0.62 ± 0.01	0.32 ± 0.12	11.77 ± 3.94
Malic acid	0.15 ± 0.02	nd	0.23 ± 0.05	nd	0.33 ± 0.14	nd
1-*O*-protocatechuoyl-glucose	nd	nd	nd	1.02 ± 0.16	nd	0.93 ± 0.04
Total	18.00 ± 2.82 [a]	7.18 ± 0.61 [c]	6.81 ± 0.91 [c]	6.14 ± 0.60 [c]	11.9 ± 1.88 [b]	18.83 ± 4.13 [a]
**Flavan-3-ols (mg EE/100g DW)**						
(Epi)catechin	0.02 ± 0.00	83.83 ± 9.70	0.02 ± 0.00	100.69 ± 6.46	0.02 ± 0.00	221.64 ± 24.73
(Epi)catechin gallate	nd	15.14 ± 3.67	nd	26.66 ± 6.38	0.04 ± 0.03	36.98 ± 7.73
(Epi)catechin hexoside	nd	3.04 ± 1.24	nd	3.48 ± 1.55	nd	5.66 ± 2.53
(Epi)gallocatechin	nd	nd	nd	0.82 ± 0.39	nd	1.07 ± 0.09
(Epi)gallocatechin gallate	nd	2.45 ± 0.29	nd	2.75 ± 0.07	nd	1.63 ± 0.16
Methyl epigallocatechin -3-gallate	0.03 ± 0.00	27.96 ± 2.16	0.04 ± 0.01	29.35 ± 4.13	0.02 ± 0.01	19.54 ± 3.30
Procyanidin A	nd	0.48 ± 0.24	nd	0.91 ± 0.24	nd	0.63 ± 0.52
Procyanidin B	nd	3.20 ± 1.96	nd	10.69 ± 3.82	nd	22.64 ± 3.06
Procyanidin trimer	nd	nd	nd	0.30 ± 0.13	nd	0.63 ± 0.13
Propelargonidin dimer	nd	0.76 ± 0.25	nd	1.20 ± 0.28	nd	2.04 ± 0.69

Supplementary Table 3 *Cont.*

Compounds [2]	Noble		Carlos		Granny Val	
	skins	seeds	skins	seeds	skins	seeds
Propelargonidin trimer	nd	nd	nd	0.64 ± 0.01	nd	nd
Total	0.05 ± 0.00 [d]	136.88 ± 16.58 [c]	0.06 ± 0.01 [d]	177.49 ± 14.13 [b]	0.09 ± 0.03 [d]	312.45 ± 32.96 [a]
**Stilbens (mg REE/100g DW)**						
Resveratrol	0.06 ± 0.00	nd	nd	nd	0.06 ± 0.00	nd
Total	0.06 ± 0.00 [a]	nd	nd	nd	0.06 ± 0.00 [a]	nd
**Cinnamic acids (mg CAE/100g DW)**						
Ferulic acid	nd	nd	0.22 ± 0.04	nd	0.59 ± 0.46	nd
Total	nd	nd	0.22 ± 0.04 [b]	nd	0.59 ± 0.46 [a]	nd

[1] Values are expressed as means of triplicate determinations ± S.D. nd, not detected. For the total concentrations, different small letters within row indicate significant differences (Duncan´s test, $p = 0.05$).

[2] Ellagic acid, (-)-epicatechin, gallic acid, kaempferol, myricetin, penta-*O*-galloyl-glucose, quercetin, resveratrol, and rutin were quantified by their standard, respectively, and expressed as mg/100g DW. GAE: gallic acid equivalent; RE: rutin equivalent; EE: (-)-epicatechin equivalent; REE: resveratrol equivalent; CAE: caffeic acid equivalent.
